# From Correlation to Causation: Defining Gene and RNA Function in Poultry Muscle Biology Using In Vivo Genetic Tools

**DOI:** 10.3390/biom15111554

**Published:** 2025-11-05

**Authors:** Bahareldin Ali Abdalla Gibril, Xuewen Chai, Jiguo Xu

**Affiliations:** Jiangxi Provincial Key Laboratory of Poultry Genetic Improvement, Institute of Biological Technology, Nanchang Normal University, Nanchang 330032, China; gibril@ncnu.edu.cn (B.A.A.G.); chaixuewen03@ncnu.edu.cn (X.C.)

**Keywords:** functional genomics, CRISPR/Cas9, viral vectors, lncRNA, circRNA, myopathies, in vivo validation, skeletal muscle

## Abstract

A central challenge in functional genomics is understanding the difference between correlative transcriptomic observations and definitive causal understanding of gene function in vivo. Poultry skeletal muscle, a system of significant agricultural and biological importance, demonstrates this challenge. While transcriptomic studies have cataloged extensive RNA expression dynamics during muscle development and in growth-related myopathies like wooden breast, establishing causative roles for these molecules is lacking. This review synthesizes how advanced genetic tools are now enabling a shift from correlation to causation in avian muscle biology. We detail how viral vectors (e.g., adenovirus, lentivirus, and RCAS) and CRISPR/Cas9 systems have provided direct in vivo validation of the functional roles of specific mRNAs, miRNAs, lncRNAs, and circRNAs in regulating myogenesis, hypertrophy, and atrophy. We contrast this success in fundamental biology with the study of myopathies, which remains largely descriptive. Here, a wealth of transcriptomic data has identified dysregulated pathways, including ECM remodeling, metabolism, and inflammation, but functional validation for most candidates is absent. We argue that the critical next step is to apply this established functional genomics toolkit to disease models. By defining causal mechanisms, this research will not only address a major agricultural issue but also provide a model for using genetic tools to dissect complex traits in a post-genomic era.

## 1. Introduction

The post-genomic era has provided researchers with an unprecedented catalog of genes and RNA transcripts whose expressions are associated with biological processes and disease states. However, a fundamental challenge persists: for the vast majority of these molecules, we lack definitive evidence of their in vivo function, struggling to distinguish causal drivers from secondary consequences [1,2]. This gap between correlation and causation is a universal problem in functional genomics. It is especially important for non-coding RNAs (ncRNAs), which are becoming more and more important in controlling development, and disease but whose mechanisms of action are often not well understood.

Poultry skeletal muscle serves as an excellent model system to address this universal challenge. It is the world’s primary source of animal protein [3] and its development is orchestrated by a complex network of coding and ncRNAs [4,5,6,7]. While the core molecular network of myogenesis is evolutionarily conserved, its timing and regulation exhibit species-specific characteristics crucial for understanding avian biology and for using poultry as a complementary model to rodents for studying rapid postnatal muscle growth. Intensive genetic selection for rapid growth has, however, triggered a high incidence of harmful muscle myopathies such as wooden breast (WB) and white striping (WS), which are characterized by fibrosis, lipidosis, and inflammation [8,9,10,11,12]. These myopathies lead to substantial economic losses for the poultry industry worldwide, primarily through meat quality degradation, increased processing waste, and whole carcass condemnation, costing the industry an estimated $200 million annually in the US alone [8]. Crucially, these pathologies are driven by widespread dysregulation of both mRNAs and ncRNAs [13,14].

This context creates a clear dichotomy in the current state of research. On one hand, the field of fundamental myogenesis has successfully used powerful in vivo tools, including species-specific viral vectors and CRISPR/Cas9 genome editing, to move from correlation to causation, defining the functional roles of specific RNAs in muscle development, hypertrophy, and atrophy in healthy birds [15,16,17]. On the other hand, the study of myopathies remains largely descriptive. While transcriptomic studies have meticulously cataloged RNA expression patterns in diseased muscle [18,19,20,21], the functional role of these dysregulated molecules is almost entirely unknown, preventing the identification of therapeutic targets.

This review aims to synthesize these two narratives through the lens of functional genomics. We first detail how genetic tools have greatly advanced our understanding of RNA-regulated muscle development in poultry, providing a causal framework for how muscle is built and maintained. We then contrast this with the current state of myopathy research, where a wealth of correlative data awaits functional examination. Finally, we highlight the urgent need and clear potential to apply this established functional toolkit to disease models as the essential next step. This will move the field beyond description and towards defining the causal mechanisms underlying these myopathies, which is a prerequisite for developing strategies to improve muscle health and sustainability.

## 2. Poultry-Specific Myogenesis: In Vivo Regulation and Development

**Figure 1:**
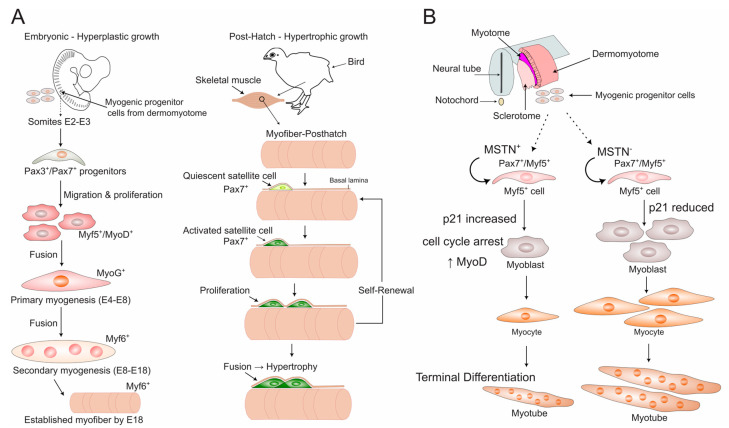
Poultry-Specific Myogenesis and its Key Molecular Regulators. (**A**) Timeline of embryonic and post-hatch muscle development in poultry. During embryogenesis, Pax3/Pax7-positive progenitor cells from the dermomyotome undergo hyperplastic growth in two waves: primary (E4–E8, marked by MyoG) and secondary (E8–E18, marked by Myf6), establishing a fixed number of myofibers by E18. Post-hatch muscle growth shifts to hypertrophy, driven by the fusion of Pax7-positive satellite cells (the adult stem cell pool) with existing myofibers. (**B**) Molecular regulation of the progenitor-to-differentiation switch by myostatin (*MSTN*). In the embryonic muscle microenvironment, MSTN signaling promotes the differentiation of Pax7/Myf5-positive progenitors into myoblasts by upregulating the cell cycle inhibitor *p21* and the myogenic determination factor *MyoD* [30]. This mechanism ensures the timely depletion of the progenitor pool for myotube formation while preserving a reservoir of cells for the satellite cell niche. Note: Abbreviations: E, embryonic day; MSTN, myostatin; Myf5/6, myogenic factor 5/6; MyoG, myogenin; Pax3/7, paired box 3/7.

Skeletal muscle development (myogenesis) in poultry is a tightly regulated process that transforms progenitor cells into contractile myofibers. This journey occurs in two primary phases (Figure 1A): a prenatal phase establishing the foundational muscle structure through fiber hyperplasia and a postnatal phase dedicated solely to growth and maintenance via fiber hypertrophy and satellite cell activity [22]. The pronounced emphasis on postnatal growth is a key characteristic of poultry myogenesis, with selection programs primarily targeting accelerated early mass gain [23]. While the core molecular network is evolutionarily conserved, its timing and regulation exhibit species-specific characteristics crucial for understanding avian biology.

### 2.1. Distinct Developmental Stages Govern Muscle Formation

The prenatal phase is one of hyperplastic growth, where both the number and type of muscle fibers are determined. In the chicken embryo, myogenesis begins in the somites, with progenitor cells giving rise to myoblasts. These myoblasts fuse to form primary myotubes, which are the first functional muscle fibers. In the limbs, this process occurs between approximately embryonic day (E)4 and E8. Following this, a separate population of myoblasts gives rise to secondary myofibers, with development continuing from E8 until E18 [24]. This complex process establishes the basic muscle architecture and determines the fixed number of muscle fibers present at hatching [25,26]. Critically, the diversity of fiber types (fast vs. fast/slow) among these primary fibers is established by distinct, committed lineages of embryonic myoblasts and occurs independently of neural innervation [27].

A critical cell population established during late embryogenesis is the satellite cells. These are quiescent myogenic precursors that originate from remaining myoblasts. Their initial abundance, determined in ovo, is a key genetic factor that critically influences the potential for all postnatal muscle growth and repair [28].

In clear contrast to the prenatal phase, post-hatch growth is exclusively hypertrophic. After hatching, no new muscle fibers are formed; instead, growth occurs purely through the enlargement of existing fibers. Satellite cells are the engines of this growth. Activated by physiological demands like mechanical load or injury, they proliferate and donate their nuclei to the existing muscle fibers, supporting protein synthesis and hypertrophy [26]. This process is regulated by a complex interplay of growth factors (e.g., *TGF-β*, *MSTN* (myostatin), *activin*, and *FGF* (fibroblast growth factor)) whose expression can vary between muscle types, influencing muscle-specific growth patterns [29]. The gradual age-related depletion of this satellite cell pool is a major factor impairing muscle repair and accelerating degeneration in adult birds [26]. The size of this quiescent paired box 7-positive (Pax7^+^) satellite cell pool is determined by a balance of signaling pathways; for instance, inhibition of MSTN signaling has been shown to expand the progenitor population by maintaining the expression of *Pax7*, a key marker of satellite cell identity and activation [30].

### 2.2. The Transcriptional Hierarchy of Avian Myogenesis

The entire process of myogenesis is orchestrated by a core set of transcription factors. The commitment of cells to the muscle lineage is initiated by *Pax3* and *Pax7*. *Pax7*, in particular, is vital for maintaining the satellite cell pool in a quiescent state and is required for their activation upon demand [28]. The myogenic program is then controlled by the muscle regulatory factors (MRFs): *Myf5*, *MyoD*, *MyoG* (myogenin), and *Myf6*. *Myf5* and *MyoD* act as determination factors, committing progenitor cells to the myogenic lineage. Following commitment, *MyoD* (often in concert with *MEF2* factors) and *MyoG* direct the terminal processes of differentiation and myoblast fusion. Finally, *Myf6* is involved in supporting the maintenance and maturation of the differentiated muscle fibers [26,31,32,33]. This precise, sequential activation of MRFs has been mapped in vivo in chicken embryos, revealing accelerated differentiation kinetics in later-forming somites and providing a foundational atlas for avian myogenesis [31,32] (Figure 1A). Recent multi-omics approaches integrating transcriptomics (RNA-seq) and chromatin accessibility (ATAC-seq) have further refined this hierarchy, identifying key temporal windows like E17 as critical for the completion of myoblast fusion and the initial establishment of muscle fiber morphology in fast- and slow-growing chicken lines [34]. While this sequential hierarchy is conserved, its precise timing and expression levels can differ between fast-growing (broiler) and slow-growing (layer) chickens, contributing to their divergent growth trajectories and muscle phenotypes [35].

The precise progression through this MRF hierarchy is not autonomous but is actively regulated by external signaling pathways within the embryonic muscle environment. A key regulator of this balance is *MSTN*, a member of the TGF-β superfamily. Using in ovo electroporation in chick embryos, Manceau et al. demonstrated that *MSTN* signaling specifically promotes the transition of Pax7^+^/Myf5^+^ muscle progenitors into terminal differentiation by activating *MyoD* expression and the cell cycle inhibitor *p21* [30] (Figure 1B). This action depletes the progenitor pool in favor of generating differentiated myofibers. Conversely, inhibiting *MSTN* signaling expands the progenitor population. This work provides direct in vivo evidence in poultry that the TGF-β pathway, via *MSTN*, is a critical external cue that modulates the core MRF network to control the balance between progenitor cell renewal and terminal differentiation during embryonic myogenesis.

## 3. Transcriptional Landscapes: RNA Dynamics in Poultry Muscle

Descriptive transcriptomic studies have been instrumental in mapping the complex RNA networks that govern poultry myogenesis. By comparing different developmental stages, breeds, and muscle types (Table S1), these studies generate crucial lists of candidate RNAs whose in vivo functions can then be probed using genetic tools. This section synthesizes these findings, highlighting conserved observations and critical knowledge gaps.

### 3.1. Developmental Time Courses Reveal Stage-Specific RNA Signatures

**Coding RNAs (mRNAs)**: Studies across chicken breeds consistently show that the expression of key myogenic genes is highly stage-specific. For instance, the fusion factor *Myomaker* peaks sharply during the critical secondary myogenesis window (E13–E15) in fast-growing broilers, directly correlating with their genetic potential for rapid growth [35]. Conversely, the negative regulator *MSTN* rises as *Myomaker* declines, and the determination factor *MyoD* shows a distinct post-hatch surge, suggesting its primary role is in postnatal hypertrophy [35,36]. Pathway analyses consistently implicate insulin signaling, ECM (extracellular matrix)–receptor interaction, and calcium signaling across studies in chickens [36,37,38] and ducks [39,40], demonstrating their fundamental roles. Multi-omics approaches are now adding an epigenetic layer, revealing how dynamic shifts in chromatin accessibility (ATAC-seq) guide these transcriptomic changes during post-hatch growth [41].

**Non-coding RNA (ncRNA)**: The expression of ncRNAs is equally dynamic and suggests complex regulatory roles. Foundational work in chicken embryos first mapped the expression of key myogenic miRNAs like miR-1 (heart and somites) and miR-206 (skeletal muscle) [42]. Subsequent sequencing studies have expanded the catalog, identifying stage-specific miRNAs associated with proliferation (e.g., miR-133a-3p at E14 in geese), differentiation (e.g., miR-206 at E19 in ducks), and metabolic shifts in post-hatch muscle [43,44,45]. Similarly, thousands of lncRNAs and circRNAs are differentially expressed across development. A common finding is the downregulation of lncRNAs linked to cell proliferation and the upregulation of those associated with metabolic processes as development proceeds [46]. circRNAs like *circSVIL* [47] and *circFBLN2* [48] show striking stage-specific expression peaks, and many harbor miRNA binding sites, suggesting roles as competitive endogenous RNAs (ceRNAs) [49].

### 3.2. Breed-Specific Signatures Underlie Divergent Growth Phenotypes

A powerful application of transcriptomics in poultry science is the identification of breed-specific RNA signatures that correlate with, and potentially drive, divergent muscle growth trajectories. These studies move beyond developmental timelines to compare genetically distinct populations, revealing the molecular basis of traits selected for in modern breeding programs.

**Coding RNA (mRNA) Divergence**: The most striking transcriptional differences emerge from comparisons between fast-growing broilers and slow-growing layers. Early microarray work established that broilers exhibit downregulation of slow-twitch fiber genes (e.g., *TNNI1*, *MB*) and satellite cell regulators (e.g., *FHL2*, *CSRP3*), suggesting a molecular compromise between rapid growth and muscle function or repair capacity [50]. This divergence begins embryonically; broilers show precocious upregulation of *IGF-I* mRNA in the pectoral muscle, driving earlier hyperplasia, while its premature expression in other muscles can paradoxically suppress differentiation, highlighting the critical importance of spatiotemporal regulation [51]. The gene *DLK1* has been consistently identified as a marker for hypertrophy, being elevated in broilers versus layers from embryo to post-hatch stages [52].

Beyond broiler-layer comparisons, studies within and between other breeds reveal signatures for other economically important traits. For instance, transcriptomics has identified genes linked to intramuscular fat deposition (e.g., *EHHADH*, *TECRL* higher in slow-growing Dagu chickens) and ECM remodeling, providing a molecular explanation for divergent meat quality attributes [53]. Perhaps the most elegant mechanism uncovered is isoform-level regulation. Kim et al. demonstrated that while total *MSTN* mRNA levels are similar, the ratio of its pro-myogenic (*MSTN-B*) to anti-myogenic (*MSTN-A*) isoform is significantly higher in broilers [54], providing a subtle but powerful regulatory mechanism for enhanced muscle growth.

Beyond protein-coding genes, ncRNAs also exhibit pronounced breed-specificity. miRNAs are frequently dysregulated between breeds. For example, miR-203 is transiently upregulated in dwarf chicken embryos, where it acts as a developmental brake by targeting proliferation (*c-JUN*) and differentiation (*MEF2C*) promoters [55]. Similarly, let-7b is upregulated in sex-linked dwarf muscle and inversely correlates with its target *IGF2BP3*, forming a potential axis for reduced growth [56]. These findings suggest that miRNAs regulate the expression of growth-related genes to create distinct phenotypes.

The regulatory landscape of other ncRNAs, including lncRNAs and circRNAs, further contributes to these breed-specific phenotypes. The lncRNA landscape also shows breed-specific patterns. *LncIRS1* was identified as upregulated in fast-growing broilers and operates as a ceRNA to sponge miR-15 family miRNAs and activate the pro-growth IGF1-PI3K/AKT pathway [15]. Similarly, circRNAs like *circITSN2* and *circLRRFIP1* are significantly upregulated in fast-growing broiler embryos compared to layers, suggesting their involvement in driving accelerated myogenesis [57,58].

**A Unifying Finding and Critical Gap**: A consistent finding across all these studies is the enrichment of specific pathways, namely, MAPK signaling, calcium signaling, insulin signaling, and ECM–receptor interaction, in the differential growth and metabolism of various breeds [53,59,60,61] (Table S1). However, the critical barrier remains: while these correlative studies are excellent for generating hypotheses, the in vivo functional validation of these breed-specific candidate RNAs is almost entirely lacking. Establishing causal relationships is the essential next step for using these signatures in genetic improvement programs.

## 4. In Vivo Methodologies for Functional Validation in Poultry Muscle

**Figure 2:**
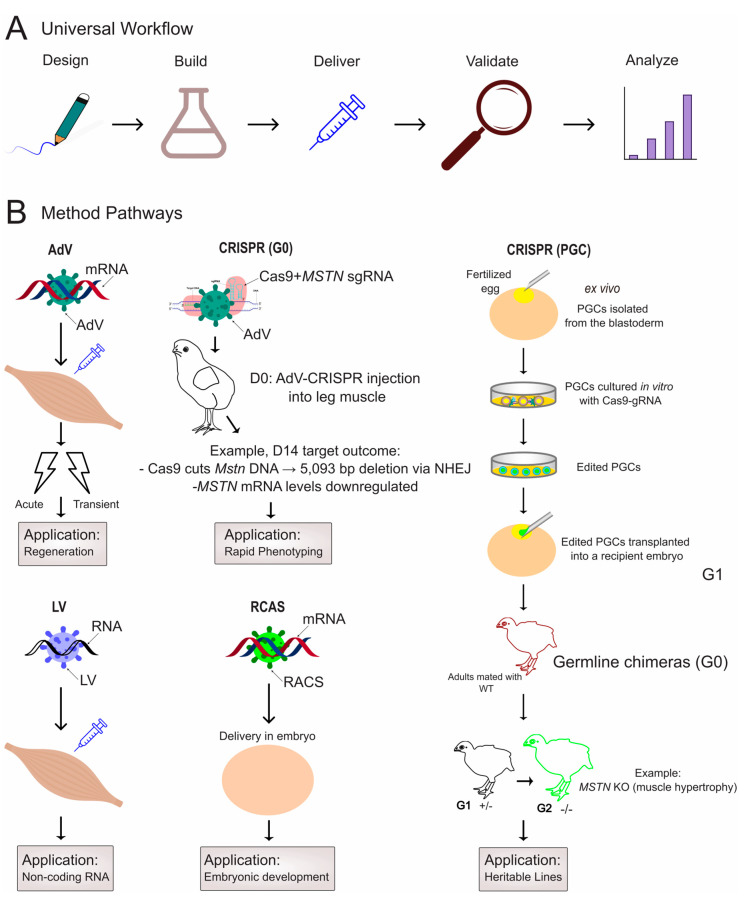
A framework for designing in vivo functional validation studies in poultry muscle. (**A**) The universal workflow for functional genomics studies, from initial design to phenotypic analysis. Icons represent key concepts: syringe for delivery, magnifying glass for validation, etc. Approximate timeframes for analysis typically range from days (acute viral expression) to weeks (CRISPR phenotyping) or months (heritable line establishment). (**B**) Method-specific operational paths for the five primary techniques, ordered alphabetically by method (AdV, CRISPR [G0 and PGC], LV, RCAS). Pathways are illustrated with schematic representations of their primary delivery targets (e.g., egg for embryonic manipulation, muscle for postnatal injection) and example applications and out-comes, such as myostatin (*MSTN*) knockout. Note: Abbreviations: AdV, adenovirus; CRISPR (G0), somatic CRISPR editing generating G0 mosaic individuals; CRISPR (PGC), primordial germ cell-mediated CRISPR for heritable editing; D, post-hatch day; gRNA, guide RNA; KO, knockout; LV, lentivirus; RCAS, replication-competent avian sarcoma-leukosis virus; WT, wild type.

Moving from correlative transcriptomic studies to establishing causative relationships requires effective methods for manipulating gene expression in vivo. Over the past decade, avian research has established a powerful toolkit for causal inference, primarily based on viral vectors and CRISPR/Cas systems (Figure 2). These technologies enable direct in vivo gain- and loss-of-function studies, allowing researchers to move beyond correlation and definitively test the functional roles of candidate genes and RNAs identified through omics approaches. The key studies developing and applying these viral and CRISPR-based tools typically employ appropriate controls (e.g., empty vector, scrambled shRNA (short hairpin RNA)) and biological replicates. Researchers should note that efficiency can vary based on delivery route, age of the animal, and specific viral batch. The selection of an appropriate method is critical and depends on several factors, including the biological question, target cell type, and the required timing and duration of manipulation (Table 1). Although physical methods like electroporation remain valuable for embryonic studies [62], and nanoparticle-based delivery represents an emerging non-viral alternative, this section will focus on viral and CRISPR-based systems due to their versatility across all developmental stages.

### 4.1. Viral Vector Delivery

Viral vectors are engineered viruses that deliver genetic material into host cells without causing disease. Their utility in poultry muscle research is well-documented, providing a versatile platform for both gain-of-function and loss-of-function studies (Table 2 and Table S2).

#### 4.1.1. Adenovirus: Efficient Transient Expression

Adenoviral vectors provide high-efficiency, high-level transient expression, making them ideal for acute interventions and studies in older animals (Table 1). A key application is the functional dissection of specific splice variants. For instance, adenovirus was used to express individual *LDB3* isoforms, revealing that they have directly opposing roles in regulating myoblast proliferation, differentiation, and regeneration [63]. Adenovirus is frequently used in muscle injury models (e.g., BaCl_2_-induced damage). It has been used to show that *RRM2* overexpression inhibits regeneration [64], while *CHAC1* (ChaC glutathione-specific gamma-glutamylcyclotransferase-1) accelerates it [65]. It also effectively demonstrates pro-growth effects, as with *VGLL2* (vestigial-like) [66] and *lncMGR* [67].

#### 4.1.2. Lentivirus: Versatility for Postnatal Manipulation

Lentiviral vectors are prized for their ability to infect non-dividing cells, such as myofibers, and provide stable, long-term expression (Table 1). These seminal studies, which we summarize in Table 2 and Table S2, established the protocol using appropriate controls (e.g., GFP-only virus) and included biological replicates to ensure reliable conclusions. Seminal work in Japanese quail established the efficacy of direct intramuscular lentiviral injection, showing high transduction efficiency even in aged or hypertrophying muscle, boosted by the use of polybrene [68]. Lentiviruses have been instrumental in dissecting the function of protein-coding genes. For example, they confirmed that the splicing factor TRA2B produces isoforms with opposing roles in regulating muscle fiber size [69] and that *PPARGC1A* (PPARG coactivator-1-alpha) overexpression drives fast-to-slow fiber switching and hypertrophy [70].

Crucially, lentiviral-mediated manipulation has been essential for defining the in vivo functions of ncRNAs: *LncIRS1:* Knockdown exacerbated atrophy, while overexpression promoted muscle growth via the IGF1-PI3K/AKT pathway [15]. *LncEDCH1:* Knockdown impaired fatty acid oxidation and induced atrophy [17]. *circGPD2:* Knockdown reduced postnatal muscle growth, proving its necessity [71]. *circAGO3:* Overexpression induced inflammatory atrophy [72]. This approach can also modulate disease states. Knocking down fibromodulin (*Fmod*) increased atrophy markers, while overexpressing it enhanced regeneration, validating its role in muscle health [73].

#### 4.1.3. RCAS Retrovirus: A Key Tool for Embryonic Studies

The Replication-Competent Avian Sarlea-Leukosis virus (RCAS) system is uniquely suited for chicken embryos due to its species specificity and infectious spread (Table 1). This tool has been foundational in understanding developmental signaling pathways. Early foundational studies used it to misexpress signaling molecules, revealing that *BMP-4* (bone morphogenetic protein 4) from the neural tube induces Wnt expression to guide myotome patterning [74] and that inhibiting BMP signaling disrupts somite development [75]. Furthermore, RCAS-mediated expression of a dominant-negative fibroblast growth factor receptor 1 (*dnFGFR1*) caused a severe reduction in embryonic muscle mass (~30%), providing direct in vivo evidence for the critical role of FGF signaling in maintaining myoblast populations [76]. In contrast, *FGF5* (fibroblast growth factor 5) overexpression inhibited myogenesis and expanded fibroblasts, underscoring the complex, context-dependent effects of FGF signaling [77]. The utility of viral delivery is further enhanced when combined with other techniques; for example, coupling in ovo electroporation with RCAS to target somites demonstrated that *MSTN* promotes terminal differentiation at the expense of the progenitor pool [30].

**Table 2:** Comparison of Selected In Vivo RNA Manipulation Techniques in Poultry Muscle Using Viral Vectors.

Poultry Type (Age)	Method (Dose)	Target RNA	Target Tissue	Duration	Key Finding/Outcome	Ref.
	**AdV**					
Chicken (D1)	AdV OV (6 × 10^8^ PFU)	mRNA; *VGLL2*	Lateral GAS	Injected once, analyzed 7D post-injection	↑ muscle fiber diameter, ↑ daily weight gain, ↑ *MyoD*, *MyoG*, *Myomaker*	[66]
Chicken (D21)	AdV OV (6 × 10^6^ PFU)	mRNA; *CHAC1*	GAS	Injected once, analyzed 1-7 D post-injury	↑ muscle fiber diameter, ↑ CSA, ↑ regeneration markers (aMyHC, eMyHC, Desmin)	[65]
	**LV**					
Chicken (D1)	LV OE (10^6^ titers)	mRNA; *PPARGC1A*	GAS	Injected at 1, 7, 14 D; phenotypic assessment at D21	↑ mitochondria, ↑ fatty acid oxidation, fast → slow shift, ↑ muscle mass	[70]
Chicken (D1)	LV OV (1 × 10^6^ IU)	mRNA; *c-Myc*-Δ269–277	Breast muscle	Two injections over 14D	miRNAs/lincRNAs dysregulation → Hypertrophy	[4]
Quail (Fertilized eggs; 4-h)	LV OV (2–3 uL)	mRNA; *MSTN-B*	Subgerminal space	42D	↑ muscle fiber hyperplasia (leg)	[78]
Chicken (D1)	LV OV (10^6^ TU)	lncRNA; *LncRNA-TBP*	Bilateral GAS	2 doses; analyzed 13D after the initial injection	↑ slow-twitch fibers, ↓ fat deposition, ↑ muscle hypertrophy via TBP recruitment	[79]
Chicken (D1)	LV OV (1 × 10^7^ TU per injection)	circRNA; *circMEF2A1/2*	Breast muscle	2 doses at D1 and D8; Tissue harvest (14D post-first injection)	↑ breast muscle mass, ↑ muscle/body weight ratio, ↑ myofiber CSA	[80]
	**RCAS**					
Chick embryo (E4)	RCAS retrovirus (2 × 10^4^ CFU)	mRNA; *IGF1*	Hindlimb mesoderm	Analyzed D3-D7 post-injection	↑ myoblast number, ↑ myofiber hyperplasia, ↑ Muscle mass	[81]

Note: Abbreviations: AdV, adenovirus; CFU, colony forming units; CHAC1, ChaC glutathione-specific gamma-glutamylcyclotransferase-1; c-Myc, v-myc avian myelocytomatosis viral oncogene homolog; circRNA, circular RNA; CSA, cross-sectional area; D, post-hatch day; GAS, gastrocnemius muscle; h, hour; IGF1, insulin-like growth factor-1; IU, infectious units; lncRNA, long non-coding RNA; LV, lentivirus; MSTN-B, myostatin isoform B; OE, overexpression; OV, overexpression vector; PFU, plaque-forming units; PPARGC1A, PPARG coactivator-1-alpha; RCAS-R, RCAS retrovirus; TU, transduction unit; uL, microliter; VGLL2, vestigial-like; →, leads to; ↓, decrease; ↑, increase.

### 4.2. CRISPR/Cas Systems: Precision Genome Editing for Functional Genomics

Complementing viral-mediated gene expression, the advent of CRISPR/Cas9 technology has transformed functional genomics by enabling precise, targeted modifications to the genome. Its application in poultry research has moved the field beyond correlation, enabling direct causal testing of gene function through knockout and targeted mutagenesis.

#### 4.2.1. PGC-Mediated Editing for Heritable Modifications

The most effective method for creating stable, heritable genome-edited poultry lines involves manipulating primordial germ cells (PGCs). A landmark study by Kim et al. demonstrated the power of this approach [82]. They used a D10A-Cas9 nickase to generate *MSTN*-knockout chickens. By co-injecting paired guide RNAs (gRNAs) into PGCs, they achieved high germline transmission rates (27–57%) and confirmed *MSTN*’s role as a potent negative regulator of muscle growth in vivo. The resulting birds exhibited significant muscle hyperplasia and reduced fat deposition, providing a clear validation of *MSTN* function and a model for enhancing muscle yield.

#### 4.2.2. Somatic Editing for Direct Phenotypic Analysis

While PGC editing creates permanent lines, somatic editing allows for direct functional testing in the same generation, bypassing the need for lengthy breeding programs.

**In ovo Somatic Editing**: This involves injecting editing reagents directly into early embryos. Lee et al. were the first to use an adenoviral method in quail by injecting an adenovirus encoding Cas9 and *MSTN*-targeting gRNAs into the blastoderm [16], resulting in a high mutation rate in founder germlines, enabling the production of homozygous mutants which exhibited significant muscle hyperplasia. A simpler, non-viral approach involves injecting plasmid DNA, as demonstrated by Huang et al. in targeting the *AMPD1* gene in chickens [83]. However, this method often results in mosaic animals, with reported editing efficiencies in target tissues ranging from 10% to 60%.

**Postnatal Somatic Editing**: CRISPR/Cas9 can be applied after hatching. Xu et al. showed that intramuscular injection of an adenoviral CRISPR vector into newborn chicks successfully knocked out *MSTN* and altered the local muscle transcriptome [84], proving the feasibility of direct, in vivo functional genomics in postnatal poultry.

**Critical Obstacles and Practical Considerations**: Despite its promise, applying CRISPR in poultry faces challenges. Editing efficiency, mosaicism in somatic editing, and delivery efficiency to target tissues remain hurdles. The choice of method depends on the research goal: PGC editing for stable lines, adenoviral in ovo editing for high efficiency in founders, and postnatal editing for acute functional studies in mature tissue.

## 5. Key RNA-Regulated Pathways in Muscle Development

**Figure 3:**
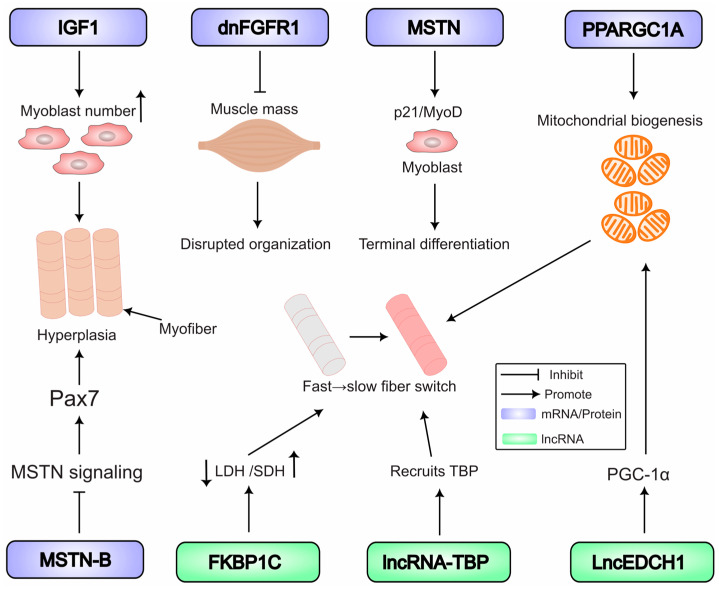
In vivo functional roles of coding and ncRNAs in poultry skeletal muscle development. Key pathways and functional outcomes validated in poultry models are summarized. Manipulation of coding RNAs: Insulin-like growth factor-1 (*IGF1*) promotes myoblast proliferation and hyperplasia; A dominant-negative fibroblast growth factor receptor 1 (*dnFGFR1*) reduces muscle mass; myostatin (*MSTN*) signaling promotes progenitor differentiation; and PPARG coactivator-1-alpha (*PPARGC1A*) overexpression drives mitochondrial biogenesis and a fast-to-slow fiber-type switch. Manipulation of non-coding RNAs: The long non-coding RNA *TBP* (*lncRNA-TBP*) and *FKBP1C* promote slow-twitch fiber characteristics. *FKBP1C* increases oxidative metabolism (succinate dehydrogenase, SDH) and inhibits glycolytic metabolism (lactate dehydrogenase, LDH). Note: Abbreviations: dnFGFR1, dominant-negative fibroblast growth factor receptor 1; IGF1, insulin-like growth factor 1; LDH, lactate dehydrogenase; lncRNA, long non-coding RNA; MSTN, myostatin; MRFs, myogenic regulatory factors; PPARGC1A, PPARG coactivator-1-alpha; SDH, succinate dehydrogenase.

The development of poultry skeletal muscle is a complex process coordinated by an intricate network of signaling pathways. The precise expression of specific coding RNAs (mRNAs) serves as a direct regulatory layer for many of these pathways. Functional studies using viral vectors and CRISPR have been instrumental in moving beyond correlation to establish causality, defining the in vivo roles of these mRNAs in avian myogenesis, from embryonic patterning through postnatal growth and fiber-type specification (Figure 3).

### 5.1. Coding RNAs: Masters of Myogenic Regulation

#### 5.1.1. The Core Myogenic Regulatory Network

At the heart of muscle development are the MRFs (*Myf5*, *MyoD*, *MyoG*, and *Myf6*). These transcription factors act as master switches, and their expression is itself tightly controlled by upstream signals. Functional evidence for their master regulatory role is clear; for example, ectopic expression of *Myf5* or *MyoD* in the chick neural tube via electroporation is sufficient to initiate the entire myogenic program, inducing the expression of downstream factors like *MyoG* and terminal differentiation markers like myosin heavy chain (*MyHC*), while actively repressing non-muscle developmental pathways [85]. Studies in quail show that mechanical load (stretch) can upregulate *MyoD* and *Myf6* mRNA within existing myonuclei, independent of satellite cell contribution, highlighting a direct mechanism for load-induced hypertrophy during growth phases [86].

#### 5.1.2. External Growth Factor Signals

The action of the MRF network is regulated by external growth factor signals. *IGF1* signaling is a potent positive regulator of muscle growth. RCAS retrovirus-mediated delivery of *IGF1* to the developing chick limb mesoderm provided direct in vivo evidence that it stimulates myoblast expansion and increases primary myofiber number (hyperplasia), specifically enlarging muscles without affecting other tissues [81]. The FGF pathway is essential for maintaining the myoblast pool. Disrupting this pathway in vivo via RCAS-mediated *dnFGFR1* expression caused a significant (~30%) loss of embryonic muscle mass and disrupted myofiber organization, proving its critical role in myoblast survival and proliferation [76]. In chick embryos, the misexpression of the Notch ligand Delta1 specifically downregulated *MyoD* mRNA, which effectively blocked the transition from proliferation to differentiation and inhibited muscle formation [87].

#### 5.1.3. The TGF-β Superfamily in Development

This superfamily contains potent regulators of embryonic and postnatal myogenesis. *MSTN*, beyond its well-characterized role as a negative regulator of postnatal muscle mass, *MSTN* plays a crucial role in embryonic development. Using in ovo electroporation in chick embryos, Manceau et al. demonstrated that *MSTN* signaling promotes the terminal differentiation of embryonic muscle progenitors by activating the expression of *p21* and *MyoD*, thereby critically regulating the balance between the progenitor pool and differentiated muscle fibers [30]. The *MSTN* gene produces multiple splice variants. The *MSTN*-B isoform acts as an endogenous inhibitor by binding to and neutralizing full-length *MSTN*, and its lentiviral overexpression in quail eggs increased muscle fiber hyperplasia by prolonging *Pax7* expression during embryogenesis [78].

#### 5.1.4. Metabolic Regulator

Lentiviral overexpression of *PPARGC1A* in early postnatal chickens drives mitochondrial biogenesis, enhances fatty acid oxidation, and induces a fast-to-slow myofiber shift, increasing muscle mass [70].

### 5.2. ncRNAs: The Sophisticated Regulators of Myogenesis

Beyond protein-coding genes, a complex layer of regulation is orchestrated by ncRNAs. These molecules modulate gene expression networks with high precision during muscle development.

#### 5.2.1. miRNAs: Master Post-Transcriptional Repressors in Development

MicroRNAs (miRNAs) are short ncRNAs that typically bind to the 3′ untranslated region (UTR) of target mRNAs to induce their degradation or translational repression. The fundamental regulation of muscle-specific miRNAs themselves has been demonstrated; electroporation of *Myf5* and *MyoD* expression constructs into the chicken neural tube was sufficient to induce ectopic expression of miR-1 and miR-206, establishing a direct link between master regulatory transcription factors and ncRNAs in the embryo [31]. A key feature of miRNA regulation is its compartmentalization. For example, modulating miR-22-3p altered the expression of its target *KLF* genes in the chicken liver but had no effect in the pectoral muscle, where these targets are not expressed [88].

#### 5.2.2. lncRNAs: Versatile Architects of Developmental Regulation

Long non-coding RNAs (lncRNAs) are a diverse class of transcripts >200 nucleotides long that regulate gene expression through various mechanisms during myogenesis. Lentiviral overexpression of *lncRNA-TBP* recruits TBP to promote slow-twitch fiber formation and muscle hypertrophy [79], while *FKBP1C* overexpression also enhances slow-twitch fibers and fiber hypertrophy [89]. *LncEDCH1* interacts with the SERCA2 pump to enhance calcium handling, activating PGC-1α in vivo and promoting mitochondrial biogenesis, thereby promoting slow-twitch fibers [17].

#### 5.2.3. circRNAs: Stable Regulators in Postnatal Growth

Circular RNAs (circRNAs) are a class of ncRNAs formed by back-splicing, making them highly stable. Their primary characterized function is as efficient miRNA sponges. Lentiviral knockdown of *circGPD2* in vivo significantly reduced breast muscle mass and myofiber size in broiler chicks, demonstrating its necessity for postnatal growth. It functions by sponging miR-203a to derepress the pro-myogenic factors *c-JUN* and *MEF2C* [71]. The *circMEF2A1/2* molecules promote muscle growth by sponging miR-30a-3p and miR-148a-5p, respectively, and form a positive feedback loop with their host gene, *MEF2A* [80].

## 6. Atrophy and Hypertrophy: RNA Networks in Muscle Mass Regulation

**Figure 4:**
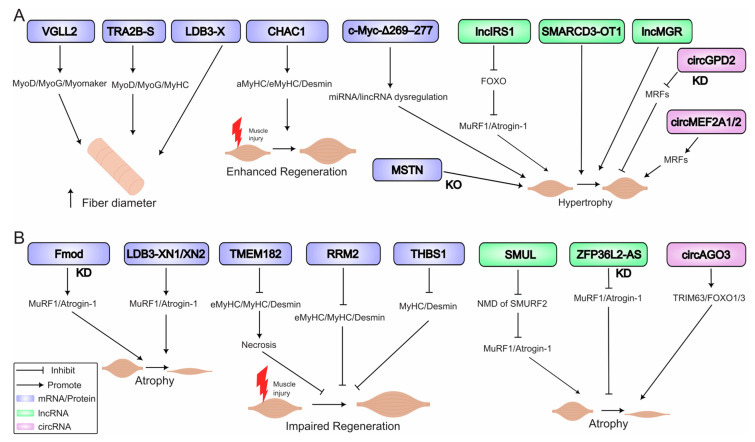
RNA networks regulating skeletal muscle hypertrophy and atrophy in poultry. (**A**) Hypertrophy drivers: Key factors promoting muscle growth include: myostatin (*MSTN*) knockout (KO); *VGLL2* increasing fiber diameter; *CHAC1* enhancing regeneration; and pro-hypertrophic long non-coding RNAs (lncRNAs) such as *lncIRS1*, *SMARCD3-OT1*, and *lncMGR*. A mutant c-Myc regulates microRNA (miRNA) and long non-coding RNA (lncRNA) networks to stimulate hypertrophy. Knockdown (KD) of circular RNA *circGPD2* also promotes hypertrophy. (**B**) Atrophy mechanisms: Key factors and processes leading to muscle wasting include: denervation and aging, which induce transcriptional reprogramming; fibromodulin (*Fmod*) knockdown, which upregulates muscle atrophy markers MuRF1 and Atrogin-1; *LDB3* isoforms (LDB3-XN1/XN2) that promote wasting; and pro-atrophic factors (*RRM2*, *TMEM182*, and *THBS1*) that impair regeneration. Knockdown of the long non-coding RNA *ZFP36L2-AS* inhibits atrophy, while the circular RNA *circAGO3* promotes muscle wasting. The nonsense-mediated mRNA decay (NMD) pathway is implicated in these regulatory networks. Note: Abbreviations: CHAC1, (ChaC glutathione-specific gamma-glutamylcyclotransferase-1); KD, knockdown; KO, knockout; lncRNA, long non-coding RNA; lncRNA, long non-coding RNA; miRNA, microRNA; MRFs, myogenic regulatory factors (MyoD, Myf5, MyoG); MSTN, myostatin; NMD, nonsense-mediated mRNA decay; PPARGC1A, PPARG coactivator-1-alpha; RRM2, ribonucleotide reductase regulatory subunit-M2; THBS1, thrombospondin 1; TMEM182, transmembrane protein 182.

The balance between muscle atrophy (wasting) and hypertrophy (growth) is dynamic and central to poultry muscle biology, affecting both meat yield and animal health. In vivo functional genomics studies have begun to elucidate the complex RNA-regulated networks that control this balance in muscle after post hatch, revealing both conserved pathways and avian-specific mechanisms (Figure 4).

### 6.1. Molecular Triggers of Atrophy

Muscle atrophy in poultry can be triggered by diverse stimuli including disuse, aging, disease states, and pharmacological interventions, characterized by the activation of specific transcriptional programs and protein degradation systems. Denervation in chickens induces a severe atrophic response, marked by a reversion to a neonatal transcriptional state, including re-expression of *MyoD*, *MyoG*, *Myf6*, and neonatal isoforms like β-tropomyosin [90]. Aging also impairs the hypertrophic response; in old quail, stretch-induced upregulation of *MyoG* mRNA is blunted due to reduced satellite cell activity [86]. Active cell death pathways contribute to atrophy beyond failed regeneration. Wing unloading in quail following hypertrophy led to elevated expression of *Id2* mRNA, which strongly correlated with increased apoptosis markers [91]. A key hallmark of atrophy is the upregulation of ubiquitin ligases like *Atrogin-1* and *MuRF-1*. Lentiviral knockdown of *Fmod* increased these atrophy markers in vivo, while its overexpression enhanced muscle mass [73]. In WB, elevated expression of myogenic regulators (*MyoD*, *MyoG*) alongside fibrotic genes indicates a failed regenerative attempt [9]. Adenoviral overexpression of *THBS1*, a target of the lncMPD2/miR-34a-5p axis, inhibits muscle regeneration after injury, reducing fiber diameter and expression of key structural markers like MyHC and Desmin [6]. Similarly, lentiviral overexpression of *TMEM182* inhibits regeneration, increasing necrosis markers and reducing fiber diameter [92]. Recent work highlights precise control of atrophy through alternative splicing; specific isoforms of *LDB3* have opposing roles: adenoviral overexpression showed *LDB3*-XN1/XN2 exacerbate wasting, while *LDB3*-X suppresses *MuRF1* expression in vitro and increases myofiber diameter (hypertrophy) in vivo [63].

ncRNAs such as the lncRNA *SMUL* promotes atrophy by triggering nonsense-mediated decay of *SMURF2* mRNA, activating the TGF-β/SMAD pathway [93]. *ZFP36L2*-AS suppresses oxidative metabolism; its lentiviral knockdown increased muscle mass and slow-twitch fibers [94]. The *circAGO3* in vitro functions as a sponge for miR-34b-5p, leading to upregulation of *TRAF3* and activation of NF-κB signaling. In vivo, lentiviral overexpression of *circAGO3* in breast muscle increased muscle atrophy markers (e.g., *TRIM63*, *FOXO1*/*3*) and decreased muscle marker genes (e.g., *MyoD*, *MyoG*, *Myf5*, and *MyHC*), while decreasing myofiber area, breast muscle weight and body weight leading to muscle wasting [72].

### 6.2. Drivers of Hypertrophy

Hypertrophy results from a positive balance between protein synthesis and degradation, driven by anabolic signals, satellite cell activity, and metabolic reprogramming. The role of *MSTN* as a potent negative regulator has been definitively proven. CRISPR/Cas9-mediated knockout induces significant hyperplasia and hypertrophy in chickens and quail [16,82]. Lentiviral overexpression of a stable *c-Myc* mutant (*c-Myc-Δ269–277*) induces hypertrophy by dysregulating miRNA and lincRNA expression networks, illustrating how coding genes orchestrate entire regulatory programs to control muscle growth [4]. Adenoviral overexpression of *VGLL2* confirmed its role in promoting postnatal muscle fiber hypertrophy, increasing fiber diameter and daily weight gain while upregulating *MyoD*, *MyoG*, and *Myomaker* expression [66]. The functional consequence of alternative splicing is a critical layer of regulation. Lentiviral overexpression of different TRA2B isoforms revealed that *TRA2B*-S promotes muscle fiber size and MRF expression, while *TRA2B*-L has opposite effects, demonstrating isoform-specific roles in myogenesis [69].

Some coding/ncRNA have been shown to be involved as pro-hypertrophic and/or promoting regeneration. For example, adenoviral overexpression of *CHAC1* enhances muscle regeneration, increasing fiber diameter, cross-sectional area, and expression of regeneration markers including aMyHC, eMyHC, and Desmin [65]. *lncIRS1* attenuates dexamethasone-induced atrophy by suppressing the FOXO-Atrogin-1/MuRF-1 pathway [15]. *SMARCD3-OT1* drives hypertrophy [95], and *lncMGR* directly promotes myofiber growth and regeneration [67]. Lentiviral overexpression of *TMEM182* inhibited muscle regeneration and induced atrophy [92], while adenoviral knockdown of *RRM2* impaired regeneration and reduced myofiber diameter [64].

### 6.3. Therapeutic Perspectives and Future Directions

The proven efficacy of manipulating key nodes in these networks, such as knocking out *MSTN* or overexpressing *lncIRS1* or knocking down *ZFP36L2-AS*, provides a strong foundation for RNA-based strategies to improve muscle mass. Future work should focus on targeting pro-inflammatory pathways like NF-κB and testing combinatorial approaches to synergistically promote growth and prevent atrophy in agricultural production.

## 7. Bridging the Functional Gap: Applying Genetic Tools to Unravel Poultry Myopathies

The growth-related myopathies WB and WS represent a critical challenge to poultry welfare and production. While transcriptomic studies have been extensive in characterizing these diseases, they have primarily generated lists of correlative associations. This section will not re-catalog these dysregulated RNAs (extensively summarized in Table S3) but will instead synthesize the conserved molecular signatures into testable hypotheses and outline a functional genomics roadmap to definitively move from correlation to causation.

### 7.1. Conserved Molecular Signatures as a Basis for Functional Inquiry

Despite differences in gross pathology, transcriptomic analyses reveal striking similarities in the molecular pathways dysregulated in WB and WS, pointing to a shared etiology centered on metabolic and structural failure. These consistent signatures provide the highest-priority candidates for functional validation: ECM Remodeling and Fibrosis: A universal finding is the significant upregulation of genes involved in extracellular matrix (ECM) synthesis and remodeling. These include fibrillar collagens such as *COL1A1*, *COL6A3*, and *COL12A1*; matrix metalloproteinases (*MMP2* and *MMP9*); and glycoproteins like *FN1* (fibronectin-1) and *SDC4* (syndecan-4) [13,18,19,96,97,98,99,100]. This presents a clear hypothesis: that the overexpression of these ECM components is a primary driver, not a secondary consequence, of fibrosis. Metabolic Dysregulation and Hypoxia: Myopathic muscle exhibits a shift in metabolic programming, with strong evidence for impaired oxidative metabolism (downregulation of *PPARGC1A/B*) and a glycolytic shift, coupled with markers of hypoxia (*HIF1A*) and altered vascular function [10,20,101,102,103]. This suggests that targeting key metabolic nodes could rescue muscle health. Inflammation and Immune Activation: A strong inflammatory signature is a hallmark of advanced disease, with upregulation of pro-inflammatory cytokines (*IL1B*, *IL6* and *TNF*), Toll-like receptors (*TLR2/4*), and activation of the NF-κB pathway [11,104]. The critical question remains whether this inflammation initiates pathology or is a response to tissue damage. Dysregulated Lipid Handling: Evidence of aberrant lipid metabolism is evident early, with upregulation of lipid uptake and storage genes (*LPL* {lipoprotein lipase}, *FABP4* {fatty acid binding protein 4 (also known as *aP2*—adipocyte protein-2)}, *PLIN1* {perilipin-1}) in affected muscle [98,105,106]. This implies that intracellular lipid accumulation may be a causative event in the disease cascade.

**Altered Satellite Cell Dynamics and Regenerative Failure**: While a depletion or dysfunction of satellite cells might be expected in a degenerative condition, the molecular profile often indicates an active, albeit unsuccessful, regenerative response. Factors that increase the expression of *Pax7*, a key marker of satellite cell identity and activation, can include signaling pathways that inhibit differentiation (e.g., *MSTN* inhibition) to maintain the progenitor pool. However, in the context of WB, transcriptomic data show that this activation is dysregulated, with studies reporting an upregulation of myogenic regulatory factors like *MyoD* and *MyoG* [107]. This is interpreted as a failed attempt at regeneration, where Pax7^+^ satellite cells are activated but cannot successfully complete the repair and differentiation process due to the severe fibrotic microenvironment and inhibitory growth factors, leading to the characteristic mix of regenerative and degenerative signals.

### 7.2. A Functional Genomics Roadmap for Myopathy Research

The pressing future direction is unequivocal: the field must pivot from descriptive cataloging to targeted in vivo functional studies. The established toolkit detailed in Section 4 is directly applicable to this task. We propose a strategic roadmap focused on validating the most promising candidate drivers (Table 3).

To implement this roadmap, specific, feasible experiments can be designed using the tools at hand: To test if miR-155 is a causal driver of fibrosis, one could inject a lentiviral sponge vector expressing multiple high-affinity miR-155 binding sites into the pectoralis major of young chicks. A resulting reduction in collagen deposition and inflammatory markers would confirm its causative role. To determine if *FABP4*-driven lipidosis is an early trigger, an adenoviral CRISPR-Cas9 system could be injected in ovo to knockout *FABP4* in the developing breast muscle. Monitoring these birds for lipid accumulation and myopathy development would test its primacy in the pathological cascade. To validate the functional impact of a non-coding RNA like a circRNA or lncRNA consistently upregulated in WB (e.g., one sponging a tumor suppressor miRNA), lentiviral-mediated overexpression would be the direct tool to recapitulate the disease-associated state and observe if it is sufficient to induce aspects of the pathology.

### 7.3. Future Directions: Integrating Functional Data into a Systems-Level Understanding

The ultimate goal is not merely to validate individual candidates but to understand the interconnected network of disease causation. Future research must focus on:

Systematic Functional Screening: The top candidates from transcriptomic studies (Table 3) must be systematically manipulated in vivo using the proposed strategies to establish a hierarchy of pathological importance.

**From GWAS to Mechanism**: Establishing causal links between genetic variants identified in genome-wide association studies (GWAS) [108], their effect on RNA expression, and ultimate disease susceptibility through genome editing is essential for genetic improvement.

**Multi-Omics Integration**: The functional data generated from these validation experiments should be integrated with transcriptomic, proteomic, and metabolomic datasets. This systems biology approach, highly aligned with the scope of Biomolecules, will enable the construction of predictive network models of disease progression, identifying key regulatory hubs that could be targeted therapeutically.

**Therapeutic Translation**: Proven efficacy in manipulating key nodes (e.g., knocking out *MSTN*) provides a foundation for RNA-based or gene-editing strategies, as well as for genomic breeding through gene-assisted selection based on validated causative genes, to improve muscle health. Future work should explore targeting pro-inflammatory pathways like NF-κB and testing combinatorial approaches to synergistically promote growth and prevent atrophy. By closing this functional gap, research can move beyond describing the problem and begin to generate genuine, mechanism-based solutions, ensuring both improved animal welfare and sustainable meat production.

## 8. Conclusions and Outlook

In conclusion, this review synthesizes two contrasting narratives in poultry muscle biology. On one hand, the field has successfully employed a powerful in vivo functional genomics toolkit, encompassing viral vectors and CRISPR/Cas9 systems, to move from correlation to causation in fundamental myogenesis. We now have definitive evidence for the roles of key genes and a growing list of ncRNAs in regulating muscle development, fiber-type specification, and mass homeostasis in healthy birds. This provides a strong causal framework for how skeletal muscle is built and maintained.

In clear contrast, the study of harmful myopathies such as WB and WS remains largely descriptive. While transcriptomic studies have been extensive, consistently implicating pathways in ECM remodeling, metabolism, and inflammation, they have generated lists of correlative candidates rather than validated therapeutic targets. The direct functional testing of these candidates in vivo represents the single most critical and urgent task for the field.

Therefore, the primary outlook of this review is that the necessary tools and foundational knowledge are now in place. The essential next step is to apply this functional genomics toolkit systematically to the problem of muscle disease. Future research must focus on the following: (1) Systematic Functional Validation: Prioritizing and testing the top candidate genes and RNAs from transcriptomic studies using the strategic roadmap outlined herein. (2) Multi-Omics Integration: Future studies should employ systematic biology approaches, integrating functional genomics data with other omics layers (e.g., proteomics, metabolomics) to construct predictive network models of disease progression. This will help uncover the core regulatory hubs and inter-tissue communication pathways that govern myopathy development. (3) From Mechanism to Application: Establishing causal links between genetic variants, RNA expression, and disease susceptibility is essential. This knowledge will directly enable the development of RNA-based therapeutic strategies and inform marker-assisted selection or gene editing programs, such as the creation of *MSTN*-knockout lines for hypermuscling, to breed more resilient poultry lines. By closing this functional gap, research can transition from describing the problem to defining its mechanisms, ultimately generating genuine solutions for improving animal welfare and sustainable meat production.

## Figures and Tables

**Table 1:** A Toolkit for Causal Inference in Poultry Muscle Biology.

Method	Primary Application	Key Advantages	Key Limitations	Ideal Use Case
Adenovirus (AdV)	Acute interventions; regeneration models	High-efficiency transduction; high-level transient expression; large payload capacity.	Triggers strong immune response; expression is transient (weeks).	Muscle injury/regeneration models; acute overexpression/knockdown in postnatal birds.
CRISPR (G0) Somatic (AdV/Plasmid)	Rapid phenotyping (G0 generation)	Bypasses need for germline transmission; direct functional testing in same generation.	Mosaicism (mix of edited/unedited cells); delivery efficiency to target tissue can be variable.	Rapid validation of gene function in embryonic or postnatal muscle without creating stable lines.
CRISPR PGC Editing	Heritable modifications; stable lines	Creates stable, non-mosaic, heritable knockout/knock-in lines; precise edits.	Technically demanding; requires long breeding programs; limited to few avian species.	Generating models for agricultural traits (e.g., *MSTN*-KO for hypermuscling) and fundamental research.
Lentivirus (LV)	Postnatal muscle; ncRNA function	Infects dividing and non-dividing cells (e.g., myofibers); stable, long-term expression; larger payload.	Lower transduction efficiency in some mature tissues; integration risks.	Validating functions of lncRNAs, circRNAs, and mRNAs in postnatal growth, atrophy, and fiber-type specification.
RCAS Retrovirus	Embryonic development	Species-specific; infectious spread within embryo for widespread expression.	Only infects dividing cells; limited to avian species; smaller payload capacity.	Studying early myogenesis, somite patterning, and progenitor cell niches.

**Table 3:** Top Candidate Drivers of Poultry Myopathies and Proposed Functional Validation Strategies.

Candidate Gene/RNA	Dysregulation Pattern	Hypothesized Role	Proposed In Vivo Validation Tool	Expected Phenotypic Outcome If Hypothesis Is Correct
*FABP4*	Embryonic development	Drives early lipid accumulation and metabolic insult.	Adenoviral CRISPR-KO in ovo or postnatal lentiviral shRNA knockdown.	Reduced intramuscular lipidosis; delayed or reduced severity of WB/WS.
miR-155	Postnatal muscle; ncRNA function	Promotes fibrosis and inflammation via target repression.	Lentiviral sponge/decoy vector to sequester miR-155 in vivo.	Attenuated fibrosis (↓ collagen) and reduced inflammatory markers.
*SDC4*	Acute interventions; regeneration models	Enhances pro-fibrotic signaling and ECM organization.	Lentiviral shRNA knockdown in young broilers.	Improved muscle texture; reduced ECM deposition.
*PPARGC1A*	Rapid phenotyping (G0 generation)	Loss impairs mitochondrial function, promoting metabolic shift.	Lentiviral overexpression in breast muscle pre-onset.	Improved oxidative metabolism; resistance to metabolic stress.
*COL6A3*	Heritable modifications; stable lines	Key structural component of pathological fibrosis.	Somatic CRISPR-KO in ovo to disrupt fibril formation.	Disorganized collagen deposition; reduced muscle stiffness.

Note: Abbreviations: COL6A3, collagen type-VI alpha-3-chain; ECM, extracellular matrix; FABP4, fatty acid binding protein-4; KO, knockout; PPARGC1A, PPARG coactivator-1-alpha; SDC4, syndecan-4; shRNA, short hairpin RNA; WB, wooden breast; WS, white striping.; ↓, decrease.

## Data Availability

No new data were generated or analyzed in this study.

## Supplementary Materials

The following supporting information can be downloaded at: https://www.mdpi.com/article/10.3390/biom15111554/s1, Table S1: Summary of Landmark Transcriptomic Studies in Poultry Muscle; Table S2: Comprehensive Comparison of In Vivo RNA Manipulation Techniques in Poultry Muscle Using Viral Vectors; Table S3: Transcriptomic profiles and targeted studies of poultry myopathies. Refs. [109,110,111,112,113,114,115,116,117,118,119,120,121,122,123,124,125,126,127,128,129,130,131,132,133,134,135,136,137,138,139,140] are cited in Supplementary Materials.

## Abbreviations

BMP	Bone morphogenetic protein
Cas	CRISPR-associated protein
ceRNA	competing endogenous RNA
*CHAC1*	Glutathione-specific gamma-glutamylcyclotransferase 1
circRNAs	Circular RNAs
CRISPR	Clustered regularly interspaced short palindromic repeats
DEcircRNAs	Differentially expressed circular RNAs
DEGs	Differentially expressed genes
DEmiRNAs	Differentially expressed microRNAs
DEmRNAs	Differentially expressed mRNAs
DElncRNAs	Differentially expressed long non-coding RNAs
*dnFGFR1*	dominant-negative fibroblast growth factor receptor 1
ECM	Extracellular matrix
E	Embryonic day (e.g., E10)
GAS	gastrocnemius muscle
*IGF1*	Insulin-like growth factor 1
lncRNAs	Long non-coding RNAs
miRNAs	microRNAs
MRF	Muscle regulatory factor (e.g., MyoD, myogenin, Myf5)
*MSTN*	Myostatin
*MyHC*	Myosin heavy chain
PGCs	Primordial germ cells
*PPARGC1A*	Peroxisome proliferator-activated receptor gamma-coactivator 1-alpha
RCAS	Replication-competent avian sarcoma-leukosis virus (Subgroup A)
RNA-seq	RNA sequencing
*TGF-β*	Transforming growth factor beta
*TMEM182*	Transmembrane protein 182
*TRA2B*	Transformer-2 beta homolog
WB	Wooden breast (myopathy)
WGCNA	Weighted gene co-expression network analysis
WS	White striping (myopathy)

## References

[1] Tarpey P.S., Smith R., Pleasance E., Whibley A., Edkins S., Hardy C., O’Meara S., Latimer C., Dicks E., Menzies A. (2009). A Systematic, Large-Scale Resequencing Screen of X-Chromosome Coding Exons in Mental Retardation. Nat. Genet..

[2] Guttman M., Amit I., Garber M., French C., Lin M.F., Feldser D., Huarte M., Zuk O., Carey B.W., Cassady J.P. (2009). Chromatin Signature Reveals over a Thousand Highly Conserved Large Non-Coding RNAs in Mammals. Nature.

[3] OECD, Food and Agriculture Organization of the United Nations (2025). OECD-FAO Agricultural Outlook 2025–2034.

[4] Luo W., Chen J., Li L., Ren X., Cheng T., Lu S., Lawal R.A., Nie Q., Zhang X., Hanotte O. (2019). C-Myc Inhibits Myoblast Differentiation and Promotes Myoblast Proliferation and Muscle Fibre Hypertrophy by Regulating the Expression of Its Target Genes, miRNAs and lincRNAs. Cell Death Differ..

[5] Zhang G., Zhang J., Wu P., Ling X., Wang Q., Zhou K., Li P., Zhang L., Ye H., Zhang Q. (2022). Transcriptome Sequencing Analysis of circRNA in Skeletal Muscle between Fast- and Slow-Growing Chickens at Embryonic Stages. Animals.

[6] Niu Y., Zhang Y., Tian W., Wang Y., Liu Y., Ji H., Cai H., Han R., Tian Y., Liu X. (2024). The Long Noncoding RNA lncMPD2 Inhibits Myogenesis by Targeting the miR-34a-5p/THBS1 Axis. Int. J. Biol. Macromol..

[7] Gibril B.A.A., Xiong X., Chai X., Xu Q., Gong J., Xu J. (2024). Unlocking the Nexus of Sirtuins: A Comprehensive Review of Their Role in Skeletal Muscle Metabolism, Development, and Disorders. Int. J. Biol. Sci..

[8] Petracci M., Soglia F., Madruga M., Carvalho L., Ida E., Estévez M. (2019). Wooden-Breast, White Striping, and Spaghetti Meat: Causes, Consequences and Consumer Perception of Emerging Broiler Meat Abnormalities. Compr. Rev. Food Sci. Food Saf..

[9] Velleman S.G., Clark D.L. (2015). Histopathologic and Myogenic Gene Expression Changes Associated with Wooden Breast in Broiler Breast Muscles. Avian Dis..

[10] Abasht B., Papah M.B., Qiu J. (2021). Evidence of Vascular Endothelial Dysfunction in Wooden Breast Disorder in Chickens: Insights through Gene Expression Analysis, Ultra-Structural Evaluation and Supervised Machine Learning Methods. PLoS ONE.

[11] Xing T., Luo D., Zhao X., Xu X., Li J., Zhang L., Gao F. (2021). Enhanced Cytokine Expression and Upregulation of Inflammatory Signaling Pathways in Broiler Chickens Affected by Wooden Breast Myopathy. J. Sci. Food Agric..

[12] Bordini M., Soglia F., Davoli R., Zappaterra M., Petracci M., Meluzzi A. (2022). Molecular Pathways and Key Genes Associated With Breast Width and Protein Content in White Striping and Wooden Breast Chicken Pectoral Muscle. Front. Physiol..

[13] Mutryn M.F., Brannick E.M., Fu W., Lee W.R., Abasht B. (2015). Characterization of a Novel Chicken Muscle Disorder through Differential Gene Expression and Pathway Analysis Using RNA-Sequencing. BMC Genom..

[14] Zambonelli P., Zappaterra M., Soglia F., Petracci M., Sirri F., Cavani C., Davoli R. (2016). Detection of Differentially Expressed Genes in Broiler Pectoralis Major Muscle Affected by White Striping—Wooden Breast Myopathies. Poult. Sci..

[15] Li Z., Cai B., Abdalla B.A., Zhu X., Zheng M., Han P., Nie Q., Zhang X. (2019). LncIRS1 Controls Muscle Atrophy via Sponging miR-15 Family to Activate IGF1-PI3K/AKT Pathway. J. Cachexia Sarcopenia Muscle.

[16] Lee J., Kim D.-H., Lee K. (2020). Muscle Hyperplasia in Japanese Quail by Single Amino Acid Deletion in MSTN Propeptide. Int. J. Mol. Sci..

[17] Cai B., Ma M., Zhang J., Wang Z., Kong S., Zhou Z., Lian L., Zhang J., Li J., Wang Y. (2022). LncEDCH1 Improves Mitochondrial Function to Reduce Muscle Atrophy by Interacting with SERCA2. Mol. Ther. Nucleic Acids.

[18] Papah M.B., Brannick E.M., Schmidt C.J., Abasht B. (2018). Gene Expression Profiling of the Early Pathogenesis of Wooden Breast Disease in Commercial Broiler Chickens Using RNA-Sequencing. PLoS ONE.

[19] Li D., Hou T., Du X., Zhao L., Zhang L., Gao F., Xing T. (2025). Integrated Analysis of miRNA and mRNA Expression Profiles Associated with Wooden Breast Myopathy in Broiler Chickens. Int. J. Biol. Macromol..

[20] Marchesi J.A.P., Ibelli A.M.G., Peixoto J.O., Cantão M.E., Pandolfi J.R.C., Marciano C.M.M., Zanella R., Settles M.L., Coutinho L.L., Ledur M.C. (2019). Whole Transcriptome Analysis of the Pectoralis Major Muscle Reveals Molecular Mechanisms Involved with White Striping in Broiler Chickens. Poult. Sci..

[21] Phillips C.A., Reading B.J., Livingston M., Livingston K., Ashwell C.M. (2020). Evaluation via Supervised Machine Learning of the Broiler Pectoralis Major and Liver Transcriptome in Association With the Muscle Myopathy Wooden Breast. Front. Physiol..

[22] Velleman S.G., McFarland D.C., Scanes C.G. (2015). Chapter 16—Skeletal Muscle. Sturkie’s Avian Physiology.

[23] Ricklefs R.E. (1985). Modification of Growth and Development of Muscles of Poultry. Poult. Sci..

[24] Stockdale F.E. (1992). Myogenic Cell Lineages. Dev. Biol..

[25] Smith J.H. (1963). Relation of Body Size to Muscle Cell Size and Number in the Chicken1,2. Poult. Sci..

[26] Xu J., Velleman S.G. (2023). Critical Role of the mTOR Pathway in Poultry Skeletal Muscle Physiology and Meat Quality: An Opinion Paper. Front. Physiol..

[27] Theobald J., DiMario J.X. (2011). Lineage-Based Primary Muscle Fiber Type Diversification Independent of MEF2 and NFAT in Chick Embryos. J Muscle Res. Cell Motil..

[28] Wang Y., Ji H., He L., Niu Y., Zhang Y., Liu Y., Tian Y., Liu X., Li H., Kang X. (2024). Establishment and Analysis of Immortalized Chicken Skeletal Muscle Satellite Cell Lines1. J. Integr. Agric..

[29] Kocamis H., McFarland D.C., Killefer J. (2001). Temporal Expression of Growth Factor Genes during Myogenesis of Satellite Cells Derived from the Biceps Femoris and Pectoralis Major Muscles of the Chicken. J. Cell. Physiol..

[30] Manceau M., Gros J., Savage K., Thomé V., McPherron A., Paterson B., Marcelle C. (2008). Myostatin Promotes the Terminal Differentiation of Embryonic Muscle Progenitors. Genes Dev..

[31] Sweetman D., Goljanek K., Rathjen T., Oustanina S., Braun T., Dalmay T., Münsterberg A. (2008). Specific Requirements of MRFs for the Expression of Muscle Specific microRNAs, miR-1, miR-206 and miR-133. Dev. Biol..

[32] Berti F., Nogueira J.M., Wöhrle S., Sobreira D.R., Hawrot K., Dietrich S. (2015). Time Course and Side-by-Side Analysis of Mesodermal, Pre-Myogenic, Myogenic and Differentiated Cell Markers in the Chicken Model for Skeletal Muscle Formation. J. Anat..

[33] Luo W., Li E., Nie Q., Zhang X. (2015). Myomaker, Regulated by MYOD, MYOG and miR-140-3p, Promotes Chicken Myoblast Fusion. Int. J. Mol. Sci..

[34] Gu S., Huang Q., Sun C., Wen C., Yang N. (2024). Transcriptomic and Epigenomic Insights into Pectoral Muscle Fiber Formation at the Late Embryonic Development in Pure Chicken Lines. Poult. Sci..

[35] Gu S., Wen C., Li J., Liu H., Huang Q., Zheng J., Sun C., Yang N. (2022). Temporal Expression of Myogenic Regulatory Genes in Different Chicken Breeds during Embryonic Development. Int. J. Mol. Sci..

[36] Yin X., Wu Y., Zhang S., Zhang T., Zhang G., Wang J. (2021). Transcriptomic Profile of Leg Muscle during Early Growth and Development in Haiyang Yellow Chicken. Arch. Anim. Breed..

[37] Xue Q., Zhang G., Li T., Ling J., Zhang X., Wang J. (2017). Transcriptomic Profile of Leg Muscle during Early Growth in Chicken. PLoS ONE.

[38] Yin X., Fang W., Yuan M., Sun H., Wang J. (2023). Transcriptome Analysis of Leg Muscles and the Effects of ALOX5 on Proliferation and Differentiation of Myoblasts in Haiyang Yellow Chickens. Genes.

[39] Hu Z., Cao J., Zhang J., Ge L., Zhang H., Liu X. (2021). Skeletal Muscle Transcriptome Analysis of Hanzhong Ma Duck at Different Growth Stages Using RNA-Seq. Biomolecules.

[40] Cao C., Cai Y., Li Y., Li T., Zhang J., Hu Z., Zhang J. (2023). Characterization and Comparative Transcriptomic Analysis of Skeletal Muscle in Female Pekin Duck and Hanzhong Ma Duck during Different Growth Stages Using RNA-Seq. Poult. Sci..

[41] Gu S., Huang Q., Jie Y., Sun C., Wen C., Yang N. (2024). Transcriptomic and Epigenomic Landscapes of Muscle Growth during the Postnatal Period of Broilers. J. Anim. Sci. Biotechnol..

[42] Darnell D.K., Kaur S., Stanislaw S., Konieczka J.H., Yatskievych T.A., Antin P.B. (2006). MicroRNA Expression during Chick Embryo Development. Dev. Dyn..

[43] Li C., Xiong T., Zhou M., Wan L., Xi S., Liu Q., Chen Y., Mao H., Liu S., Chen B. (2020). Characterization of microRNAs during Embryonic Skeletal Muscle Development in the Shan Ma Duck. Animals.

[44] Liu J., Li F., Hu X., Cao D., Liu W., Han H., Zhou Y., Lei Q. (2021). Deciphering the miRNA Transcriptome of Breast Muscle from the Embryonic to Post-Hatching Periods in Chickens. BMC Genom..

[45] Zeng B., Tang M., Chen T., Jiang Y., Tang W., Yu G. (2025). Characterization and Analysis of MicroRNA during Leg Muscle Development in Embryonic Stage of Daozhou Grey Goose. Poult. Sci..

[46] Liu J., Zhou Y., Hu X., Yang J., Lei Q., Liu W., Han H., Li F., Cao D. (2021). Transcriptome Analysis Reveals the Profile of Long Non-Coding RNAs During Chicken Muscle Development. Front. Physiol..

[47] Ouyang H., Chen X., Li W., Li Z., Nie Q., Zhang X. (2018). Circular RNA circSVIL Promotes Myoblast Proliferation and Differentiation by Sponging miR-203 in Chicken. Front. Genet..

[48] Liu S., Wu J., Jiang H., Zhou Y., Huang X., Wang Y., Xie Z., Liao Z., Ding Z., Liu J. (2025). CircFBLN2 Regulates Duck Myoblast Proliferation and Differentiation through miR-22-5p and MEF2C Interaction. Poult. Sci..

[49] Ouyang H., Chen X., Wang Z., Yu J., Jia X., Li Z., Luo W., Abdalla B.A., Jebessa E., Nie Q. (2018). Circular RNAs Are Abundant and Dynamically Expressed during Embryonic Muscle Development in Chickens. DNA Res..

[50] Zheng Q., Zhang Y., Chen Y., Yang N., Wang X.-J., Zhu D. (2009). Systematic Identification of Genes Involved in Divergent Skeletal Muscle Growth Rates of Broiler and Layer Chickens. BMC Genom..

[51] Al-Musawi S.L., Lock F., Simbi B.H., Bayol S.A.M., Stickland N.C. (2011). Muscle Specific Differences in the Regulation of Myogenic Differentiation in Chickens Genetically Selected for Divergent Growth Rates. Differentiation.

[52] Shin J., Velleman S.G., Latshaw J.D., Wick M.P., Suh Y., Lee K. (2009). The Ontogeny of Delta-like Protein 1 Messenger Ribonucleic Acid Expression during Muscle Development and Regeneration: Comparison of Broiler and Leghorn Chickens. Poult. Sci..

[53] San J., Du Y., Wu G., Xu R., Yang J., Hu J. (2021). Transcriptome Analysis Identifies Signaling Pathways Related to Meat Quality in Broiler Chickens—The Extracellular Matrix (ECM) Receptor Interaction Signaling Pathway. Poult. Sci..

[54] Kim D.-H., Choi Y.M., Lee J., Shin S., Kim S., Suh Y., Lee K. (2022). Differential Expression of MSTN Isoforms in Muscle between Broiler and Layer Chickens. Animals.

[55] Luo W., Wu H., Ye Y., Li Z., Hao S., Kong L., Zheng X., Lin S., Nie Q., Zhang X. (2014). The Transient Expression of miR-203 and Its Inhibiting Effects on Skeletal Muscle Cell Proliferation and Differentiation. Cell Death Dis..

[56] Lin S., Luo W., Ye Y., Bekele E.J., Nie Q., Li Y., Zhang X. (2017). Let-7b Regulates Myoblast Proliferation by Inhibiting IGF2BP3 Expression in Dwarf and Normal Chicken. Front. Physiol..

[57] Shen X., Wei Y., Liu W., You G., Tang S., Su Z., Du M., He J., Zhao J., Tian Y. (2021). A Novel Circular RNA circITSN2 Targets the miR-218-5p/LMO7 Axis to Promote Chicken Embryonic Myoblast Proliferation and Differentiation. Front. Cell Dev. Biol..

[58] Wu Y., Zhao J., Zhao X., He H., Cui C., Zhang Y., Zhu Q., Yin H., Han S. (2023). CircLRRFIP1 Promotes the Proliferation and Differentiation of Chicken Skeletal Muscle Satellite Cells by Sponging the miR-15 Family via Activating AKT3-mTOR/p70S6K Signaling Pathway. Poult. Sci..

[59] Ren T., Li Z., Zhou Y., Liu X., Han R., Wang Y., Yan F., Sun G., Li H., Kang X. (2018). Sequencing and Characterization of lncRNAs in the Breast Muscle of Gushi and Arbor Acres Chickens. Genome.

[60] Zhang Z., Qiu M., Du H., Li Q., Gan W., Xiong X., Yu C., Peng H., Xia B., Song X. (2020). Small RNA Sequencing of Pectoral Muscle Tissue Reveals microRNA-Mediated Gene Modulation in Chicken Muscle Growth. J. Anim. Physiol. Anim. Nutr..

[61] Wu P., Zhou K., Zhang L., Li P., He M., Zhang X., Ye H., Zhang Q., Wei Q., Zhang G. (2021). High-Throughput Sequencing Reveals Crucial miRNAs in Skeletal Muscle Development of Bian Chicken. Br. Poult. Sci..

[62] Scaal M., Gros J., Lesbros C., Marcelle C. (2004). In Ovo Electroporation of Avian Somites. Dev. Dyn..

[63] Wei C., Niu Y., Chen B., Wang Y., Cai H., Han R., Tian Y., Liu X., Guo W., Kang X. (2024). Divergent Regulatory Roles of Transcriptional Variants of the Chicken LDB3 Gene in Muscle Shaping. J. Agric. Food Chem..

[64] Chen B., Zhang Y., Niu Y., Wang Y., Liu Y., Ji H., Han R., Tian Y., Liu X., Kang X. (2024). RRM2 Promotes the Proliferation of Chicken Myoblasts, Inhibits Their Differentiation and Muscle Regeneration. Poult. Sci..

[65] Chen B., Cai H., Niu Y., Zhang Y., Wang Y., Liu Y., Han R., Liu X., Kang X., Li Z. (2024). Whole Transcriptome Profiling Reveals a lncMDP1 That Regulates Myogenesis by Adsorbing miR-301a-5p Targeting CHAC1. Commun. Biol..

[66] Li W., Ma H., Wang Y., Zhang Y., Liu Y., Han R., Li H., Cai H., Liu X., Kang X. (2025). The VGLL2 Gene Participates in Muscle Development in Gushi Chickens. J. Integr. Agric..

[67] Guo Y., Geng W., Chen Z., Zhi Y., Zhang K., Li Z., Li G., Kang X., Tian W., Li H. (2024). LncRNA lncMGR Regulates Skeletal Muscle Development and Regeneration by Recruiting CDK9 and Sponging miRNAs. Int. J. Biol. Macromol..

[68] Ouyang J., Alway S.E. (2004). Transgene Expression in Hypertrophied and Aged Skeletal Muscle in Vivo by Lentivirus Delivery. J. Gene Med..

[69] Chen G., Chen J., Qi L., Yin Y., Lin Z., Wen H., Zhang S., Xiao C., Bello S.F., Zhang X. (2024). Bulk and Single-Cell Alternative Splicing Analyses Reveal Roles of TRA2B in Myogenic Differentiation. Cell Prolif..

[70] Ma M., Cai B., Kong S., Zhou Z., Zhang J., Zhang X., Nie Q. (2022). PPARGC1A Is a Moderator of Skeletal Muscle Development Regulated by miR-193b-3p. Int. J. Mol. Sci..

[71] Shen X., Cui C., Tang S., Han S., Zhang Y., Xia L., Tan B., Ma M., Kang H., Yu J. (2022). MyoG-Enhanced circGPD2 Regulates Chicken Skeletal Muscle Development by Targeting miR-203a. Int. J. Biol. Macromol..

[72] Zhao X., Tang S., Lei Z., Shen X., Zhang Y., Han S., Yin H., Cui C. (2024). circAGO3 Facilitates NF-κB Pathway-Mediated Inflammatory Atrophy in Chicken Skeletal Muscle via the miR-34b-5p/TRAF3 Axis. Int. J. Biol. Macromol..

[73] Yin H., Cui C., Han S., Chen Y., Zhao J., He H., Li D., Zhu Q. (2020). Fibromodulin Modulates Chicken Skeletal Muscle Development via the Transforming Growth Factor-β Signaling Pathway. Animals.

[74] Marcelle C., Stark M.R., Bronner-Fraser M. (1997). Coordinate Actions of BMPs, Wnts, Shh and Noggin Mediate Patterning of the Dorsal Somite. Development.

[75] Capdevila J., Johnson R.L. (1998). Endogenous and Ectopic Expression of Noggin Suggests a Conserved Mechanism for Regulation of BMP Function during Limb and Somite Patterning. Dev. Biol..

[76] Flanagan-Steet H., Hannon K., McAvoy M.J., Hullinger R., Olwin B.B. (2000). Loss of FGF Receptor 1 Signaling Reduces Skeletal Muscle Mass and Disrupts Myofiber Organization in the Developing Limb. Dev. Biol..

[77] Clase K.L., Mitchell P.J., Ward P.J., Dorman C.M., Johnson S.E., Hannon K. (2000). FGF5 Stimulates Expansion of Connective Tissue Fibroblasts and Inhibits Skeletal Muscle Development in the Limb. Dev. Dyn..

[78] Chen P.R., Suh Y., Shin S., Woodfint R.M., Hwang S., Lee K. (2019). Exogenous Expression of an Alternative Splicing Variant of Myostatin Prompts Leg Muscle Fiber Hyperplasia in Japanese Quail. Int. J. Mol. Sci..

[79] Ma M., Cai B., Zhou Z., Kong S., Zhang J., Xu H., Zhang X., Nie Q. (2023). LncRNA-TBP Mediates TATA-Binding Protein Recruitment to Regulate Myogenesis and Induce Slow-Twitch Myofibers. Cell Commun. Signal..

[80] Shen X., Zhao X., He H., Zhao J., Wei Y., Chen Y., Han S., Zhu Y., Zhang Y., Zhu Q. (2023). Evolutionary Conserved Circular MEF2A RNAs Regulate Myogenic Differentiation and Skeletal Muscle Development. PLoS Genet..

[81] Mitchell P.J., Johnson S.E., Hannon K. (2002). Insulin-like Growth Factor I Stimulates Myoblast Expansion and Myofiber Development in the Limb. Dev. Dyn..

[82] Kim G.-D., Lee J.H., Song S., Kim S.W., Han J.S., Shin S.P., Park B.-C., Park T.S. (2020). Generation of Myostatin-Knockout Chickens Mediated by D10A-Cas9 Nickase. FASEB J..

[83] Huang Z., Wang J., Huang Z., Tang G., Lv G., Li D., Yang C. (2024). Functional Prediction of AMP Deaminase 1 in Jingyuan Chicken and Evaluation of the Biological Activities of Its Expression Vectors. Int. J. Biol. Macromol..

[84] Xu K., Han C.X., Zhou H., Ding J.M., Xu Z., Yang L.Y., He C., Akinyemi F., Zheng Y.M., Qin C. (2020). Effective MSTN Gene Knockout by AdV-Delivered CRISPR/Cas9 in Postnatal Chick Leg Muscle. Int. J. Mol. Sci..

[85] Delfini M.-C., Duprez D. (2004). Ectopic Myf5 or MyoD Prevents the Neuronal Differentiation Program in Addition to Inducing Skeletal Muscle Differentiation, in the Chick Neural Tube. Development.

[86] Lowe D.A., Alway S.E. (1999). Stretch-Induced Myogenin, MyoD, and MRF4 Expression and Acute Hypertrophy in Quail Slow-Tonic Muscle Are Not Dependent upon Satellite Cell Proliferation. Cell Tissue Res..

[87] Delfini M.C., Hirsinger E., Pourquié O., Duprez D. (2000). Delta 1-Activated Notch Inhibits Muscle Differentiation without Affecting Myf5 and Pax3 Expression in Chick Limb Myogenesis. Development.

[88] Ma Z., Chu H., Li F., Han G., Cai Y., Yi J., Lu M., Xiang H., Kang H., Ye F. (2024). Genome-Wide Identification, Evolution, and miRNA-22 Regulation of Kruppel-Like Factor (KLF) Gene Family in Chicken (Gallus Gallus). Animals.

[89] Yu J.-A., Wang Z., Yang X., Ma M., Li Z., Nie Q. (2021). LncRNA-FKBP1C Regulates Muscle Fiber Type Switching by Affecting the Stability of MYH1B. Cell Death Discov..

[90] Saitoh O., Fujisawa-Sehara A., Nabeshima Y., Periasamy M. (1993). Expression of Myogenic Factors in Denervated Chicken Breast Muscle: Isolation of the Chicken Myf5 Gene. Nucleic Acids Res..

[91] Alway S.E., Martyn J.K., Ouyang J., Chaudhrai A., Murlasits Z.S. (2003). Id2 Expression during Apoptosis and Satellite Cell Activation in Unloaded and Loaded Quail Skeletal Muscles. Am. J. Physiol. Regul. Integr. Comp. Physiol..

[92] Luo W., Lin Z., Chen J., Chen G., Zhang S., Liu M., Li H., He D., Liang S., Luo Q. (2021). TMEM182 Interacts with Integrin Beta 1 and Regulates Myoblast Differentiation and Muscle Regeneration. J. Cachexia Sarcopenia Muscle.

[93] Cai B., Li Z., Ma M., Zhang J., Kong S., Abdalla B.A., Xu H., Jebessa E., Zhang X., Lawal R.A. (2021). Long Noncoding RNA SMUL Suppresses SMURF2 Production-Mediated Muscle Atrophy via Nonsense-Mediated mRNA Decay. Mol. Ther. Nucleic Acids.

[94] Cai B., Ma M., Zhang J., Kong S., Zhou Z., Li Z., Abdalla B.A., Xu H., Zhang X., Lawal R.A. (2022). Long Noncoding RNA ZFP36L2-AS Functions as a Metabolic Modulator to Regulate Muscle Development. Cell Death Dis..

[95] Zhang J., Cai B., Ma M., Kong S., Zhou Z., Zhang X., Nie Q. (2022). LncRNA SMARCD3-OT1 Promotes Muscle Hypertrophy and Fast-Twitch Fiber Transformation via Enhancing SMARCD3X4 Expression. Int. J. Mol. Sci..

[96] Pampouille E., Hennequet-Antier C., Praud C., Juanchich A., Brionne A., Godet E., Bordeau T., Fagnoul F., Le Bihan-Duval E., Berri C. (2019). Differential Expression and Co-Expression Gene Network Analyses Reveal Molecular Mechanisms and Candidate Biomarkers Involved in Breast Muscle Myopathies in Chicken. Sci. Rep..

[97] Pejšková L., Rønning S.B., Kent M.P., Solberg N.T., Høst V., Thu-Hien T., Wold J.P., Lunde M., Mosleth E., Pisconti A. (2023). Characterization of Wooden Breast Myopathy: A Focus on Syndecans and ECM Remodeling. Front. Physiol..

[98] Wang Z., Khondowe P., Brannick E., Abasht B. (2024). Spatial Transcriptomics Reveals Alterations in Perivascular Macrophage Lipid Metabolism in the Onset of Wooden Breast Myopathy in Broiler Chickens. Sci. Rep..

[99] Malila Y., Uengwetwanit T., Thanatsang K.V., Arayamethakorn S., Srimarut Y., Petracci M., Soglia F., Rungrassamee W., Visessanguan W. (2021). Insights Into Transcriptome Profiles Associated With Wooden Breast Myopathy in Broilers Slaughtered at the Age of 6 or 7 Weeks. Front. Physiol..

[100] Praud C., Jimenez J., Pampouille E., Couroussé N., Godet E., Le Bihan-Duval E., Berri C. (2020). Molecular Phenotyping of White Striping and Wooden Breast Myopathies in Chicken. Front. Physiol..

[101] Malila Y., Thanatsang K., Arayamethakorn S., Uengwetwanit T., Srimarut Y., Petracci M., Strasburg G.M., Rungrassamee W., Visessanguan W. (2019). Absolute Expressions of Hypoxia-Inducible Factor-1 Alpha (HIF1A) Transcript and the Associated Genes in Chicken Skeletal Muscle with White Striping and Wooden Breast Myopathies. PLoS ONE.

[102] Maharjan P., Beitia A., Weil J., Suesuttajit N., Hilton K., Caldas J., Umberson C., Martinez D., Kong B., Owens C.M. (2021). Woody Breast Myopathy Broiler Show Age-Dependent Adaptive Differential Gene Expression in Pectoralis Major and Altered in-Vivo Triglyceride Kinetics in Adipogenic Tissues. Poult. Sci..

[103] Bordini M., Wang Z., Soglia F., Petracci M., Schmidt C.J., Abasht B. (2024). RNA-Sequencing Revisited Data Shed New Light on Wooden Breast Myopathy. Poult. Sci..

[104] Lu J., Yuan H., Liu S., Liu Y., Qin Z., Han W., Zhang R. (2024). Gene Coexpression Network Analysis Reveals the Genes and Pathways in Pectoralis Major Muscle and Liver Associated with Wooden Breast in Broilers. Poult. Sci..

[105] Brothers B., Zhuo Z., Papah M.B., Abasht B. (2019). RNA-Seq Analysis Reveals Spatial and Sex Differences in Pectoralis Major Muscle of Broiler Chickens Contributing to Difference in Susceptibility to Wooden Breast Disease. Front. Physiol..

[106] Lake J.A., Papah M.B., Abasht B. (2019). Increased Expression of Lipid Metabolism Genes in Early Stages of Wooden Breast Links Myopathy of Broilers to Metabolic Syndrome in Humans. Genes.

[107] Velleman S.G. (2015). Relationship of Skeletal Muscle Development and Growth to Breast Muscle Myopathies: A Review. Avian Dis..

[108] Pampouille E., Berri C., Boitard S., Hennequet-Antier C., Beauclercq S.A., Godet E., Praud C., Jégo Y., Le Bihan-Duval E. (2018). Mapping QTL for White Striping in Relation to Breast Muscle Yield and Meat Quality Traits in Broiler Chickens. BMC Genom..

[109] Li J., Yang D., Chen C., Wang J., Wang Z., Yang C., Yu C., Li Z. (2025). Single-Cell RNA Transcriptome Uncovers Distinct Developmental Trajectories in the Embryonic Skeletal Muscle of Daheng Broiler and Tibetan Chicken. BMC Genom..

[110] Li F., Zhu C., Luo Y., Li S., Wang Q., Han Y., Wu Z., Li X., Liang Y., Chen Y. (2023). Transcriptomic Analysis on Pectoral Muscle of European Meat Pigeons and Shiqi Pigeons during Embryonic Development. Animals.

[111] Wang Z., Tian W., Wang D., Guo Y., Cheng Z., Zhang Y., Li X., Zhi Y., Li D., Li Z. (2023). Comparative Analyses of Dynamic Transcriptome Profiles Highlight Key Response Genes and Dominant Isoforms for Muscle Development and Growth in Chicken. Genet. Sel. Evol..

[112] Zhang Y., Li D., Han R., Wang Y., Li G., Liu X., Tian Y., Kang X., Li Z. (2017). Transcriptome Analysis of the Pectoral Muscles of Local Chickens and Commercial Broilers Using Ribo-Zero Ribonucleic Acid Sequencing. PLoS ONE.

[113] Sporer K.R.B., Tempelman R.J., Ernst C.W., Reed K.M., Velleman S.G., Strasburg G.M. (2011). Transcriptional Profiling Identifies Differentially Expressed Genes in Developing Turkey Skeletal Muscle. BMC Genom..

[114] Zhou K.-Z., Wu P.-F., Zhang X.-C., Ling X.-Z., Zhang J., Zhang L., Li P.-F., Zhang T., Wei Q.-Y., Zhang G.-X. (2022). Comparative Analysis of miRNA Expression Profiles in Skeletal Muscle of Bian Chickens at Different Embryonic Ages. Animals.

[115] Wu P., He M., Zhang X., Zhou K., Zhang T., Xie K., Dai G., Wang J., Wang X., Zhang G. (2022). miRNA-Seq Analysis in Skeletal Muscle of Chicken and Function Exploration of miR-24-3p. Poult. Sci..

[116] Ju X., Liu Y., Shan Y., Ji G., Zhang M., Tu Y., Zou J., Chen X., Geng Z., Shu J. (2021). Analysis of Potential Regulatory LncRNAs and CircRNAs in the Oxidative Myofiber and Glycolytic Myofiber of Chickens. Sci. Rep..

[117] Liu Y., Zhang M., Shan Y., Ji G., Ju X., Tu Y., Sheng Z., Xie J., Zou J., Shu J. (2020). miRNA-mRNA Network Regulation in the Skeletal Muscle Fiber Phenotype of Chickens Revealed by Integrated Analysis of miRNAome and Transcriptome. Sci. Rep..

[118] Li Y., Chen Y., Jin W., Fu S., Li D., Zhang Y., Sun G., Jiang R., Han R., Li Z. (2019). Analyses of MicroRNA and mRNA Expression Profiles Reveal the Crucial Interaction Networks and Pathways for Regulation of Chicken Breast Muscle Development. Front. Genet..

[119] Jebessa E., Ouyang H., Abdalla B.A., Li Z., Abdullahi A.Y., Liu Q., Nie Q., Zhang X. (2018). Characterization of miRNA and Their Target Gene during Chicken Embryo Skeletal Muscle Development. Oncotarget.

[120] Khatri B., Seo D., Shouse S., Pan J.H., Hudson N.J., Kim J.K., Bottje W., Kong B.C. (2018). MicroRNA Profiling Associated with Muscle Growth in Modern Broilers Compared to an Unselected Chicken Breed. BMC Genom..

[121] Ouyang H., He X., Li G., Xu H., Jia X., Nie Q., Zhang X. (2015). Deep Sequencing Analysis of miRNA Expression in Breast Muscle of Fast-Growing and Slow-Growing Broilers. Int. J. Mol. Sci..

[122] Li T., Wu R., Zhang Y., Zhu D. (2011). A Systematic Analysis of the Skeletal Muscle miRNA Transcriptome of Chicken Varieties with Divergent Skeletal Muscle Growth Identifies Novel miRNAs and Differentially Expressed miRNAs. BMC Genom..

[123] Rathjen T., Pais H., Sweetman D., Moulton V., Munsterberg A., Dalmay T. (2009). High Throughput Sequencing of microRNAs in Chicken Somites. FEBS Lett..

[124] Miao D., Zhang C., Wu X., Wang Y., Chowdhury V.S., Yang H., Wang Z. (2025). Transcriptomic Analysis of Long Non-Coding RNA and mRNA in Pigeon Squab Pectoralis Muscle Development. Br. Poult. Sci..

[125] Hong L., Xu D., Li W., Wang Y., Cao N., Fu X., Tian Y., Li Y., Li B. (2023). Non-Coding RNA Regulation of Magang Geese Skeletal Muscle Maturation via the MAPK Signaling Pathway. Front. Physiol..

[126] Luo Y., Hu S., Yan P., Wu J., Guo H., Zhao L., Tang Q., Ma J., Long K., Jin L. (2022). Analysis of mRNA and lncRNA Expression Profiles of Breast Muscle during Pigeon (Columbalivia) Development. Genes.

[127] Li Z., Ouyang H., Zheng M., Cai B., Han P., Abdalla B.A., Nie Q., Zhang X. (2017). Integrated Analysis of Long Non-Coding RNAs (LncRNAs) and mRNA Expression Profiles Reveals the Potential Role of LncRNAs in Skeletal Muscle Development of the Chicken. Front. Physiol..

[128] Li T., Wang S., Wu R., Zhou X., Zhu D., Zhang Y. (2012). Identification of Long Non-Protein Coding RNAs in Chicken Skeletal Muscle Using next Generation Sequencing. Genomics.

[129] Shao B., Wang Z., Luo P., Du P., Zhang X., Zhang H., Si X., Ma S., Chen W., Huang Y. (2025). Identifying Insulin-Responsive circRNAs in Chicken Pectoralis. BMC Genom..

[130] Cai B., Ma M., Zhou Z., Kong S., Zhang J., Zhang X., Nie Q. (2022). circPTPN4 Regulates Myogenesis via the miR-499-3p/NAMPT Axis. J. Anim. Sci. Biotechnol..

[131] Wu P., Zhou K., Zhang J., Ling X., Zhang X., Zhang L., Li P., Wei Q., Zhang T., Wang X. (2022). Identification of Crucial circRNAs in Skeletal Muscle during Chicken Embryonic Development. BMC Genom..

[132] Zhang W., Liu J., Zhou Y., Liu S., Wu J., Jiang H., Xu J., Mao H., Liu S., Chen B. (2024). Signaling Pathways and Regulatory Networks in Quail Skeletal Muscle Development: Insights from Whole Transcriptome Sequencing. Poult. Sci..

[133] Zhang T., Chen C., Han S., Chen L., Ding H., Lin Y., Zhang G., Xie K., Wang J., Dai G. (2021). Integrated Analysis Reveals a lncRNA-miRNA-mRNA Network Associated with Pigeon Skeletal Muscle Development. Genes.

[134] Cai B., Ma M., Yuan R., Zhou Z., Zhang J., Kong S., Lin D., Lian L., Li J., Zhang X. (2024). MYH1G-AS Is a Chromatin-Associated lncRNA That Regulates Skeletal Muscle Development in Chicken. Cell. Mol. Biol. Lett..

[135] Padilha S.F., Ibelli A.M.G., Peixoto J.O., Cantão M.E., Moreira G.C.M., Fernandes L.T., Tavernari F.C., Morés M.A.Z., Bastos A.P.A., Dias L.T. (2024). Novel Candidate Genes Involved in an Initial Stage of White Striping Development in Broiler Chickens. Animals.

[136] Marciano C.M.M., Ibelli A.M.G., Marchesi J.A.P., de Oliveira Peixoto J., Fernandes L.T., Savoldi I.R., do Carmo K.B., Ledur M.C. (2021). Differential Expression of Myogenic and Calcium Signaling-Related Genes in Broilers Affected With White Striping. Front. Physiol..

[137] Malila Y., Uengwetwanit T., Arayamethakorn S., Srimarut Y., Thanatsang K.V., Soglia F., Strasburg G.M., Rungrassamee W., Visessanguan W. (2020). Transcriptional Profiles of Skeletal Muscle Associated With Increasing Severity of White Striping in Commercial Broilers. Front. Physiol..

[138] Pizzol M.S.D., Ibelli A.M.G., Cantão M.E., Campos F.G., de Oliveira H.C., de Oliveira Peixoto J., Fernandes L.T., de Castro Tavernari F., Morés M.A.Z., Bastos A.P.A. (2024). Differential Expression of miRNAs Associated with Pectoral Myopathies in Young Broilers: Insights from a Comparative Transcriptome Analysis. BMC Genom..

[139] Shu J., Liu Y., Shan Y., Ji G., Ju X., Tu Y., Shi S., Sheng Z., Zhang M., Zou J. (2021). Deep Sequencing microRNA Profiles Associated with Wooden Breast in Commercial Broilers. Poult. Sci..

[140] Wang Z., Brannick E., Abasht B. (2023). Integrative Transcriptomic and Metabolomic Analysis Reveals Alterations in Energy Metabolism and Mitochondrial Functionality in Broiler Chickens with Wooden Breast. Sci. Rep..

