# Supplementary material for: From Correlation to Causation: Defining Gene and RNA Function in Poultry Muscle Biology Using In Vivo Genetic Tools
Source: Biomolecules. 2025 Nov 5;15(11):1554. doi: 10.3390/biom15111554 (PMC12650688; doi:10.3390/biom15111554)
Supplement: Supplementary file 1 [file biomolecules-15-01554-s001.zip › biomolecules-3946548-supplementary.pdf]

Supplementary Material

Table S1. Summary of Landmark Transcriptomic Studies in Poultry Muscle.

Poultry Type	Contrast	Tissue	Method	Seq. Tech.	Key Targets	Func. Enrichment	Ref.
Chicken	Daheng broiler vs. Tibetan chicken (E10, E14, E18)	Leg muscle	scRNA-seq	Illumina NovaSeq 6000	*SNRPG*, *SNRPE*, *EIF4EBP1* (myogenesis); *APOA1*, *MGP* (adipogenesis)	Ribosome biogenesis, muscle cell differentiation, lipid metabolism	[109]
Chicken	Cornish (CC) & White Plymouth Rock (D21 vs. D42)	Pectoralis	RNA-seq, ATAC-seq	Illumina NovaSeq 6000	↑D21: *ACTC1*, *FDPS*, *NRG1*; ↑D42: *MUSTN1*, *FOS*, *TGFB3*; Breed: *TMEM164* (CC)	Muscle structure development, cell differentiation, cholesterol metab., immune response	[41]
Pigeon	European meat vs. Shigi (E6, E10, E14, D1)	Pectoralis	RNA-seq	Illumina NovaSeq 6000	*CLU*, *PTGS1*, *NR1H4*, *MYH6*	Calcium signaling, PPAR pathway, ECM-receptor interactions	[110]
Chicken	E12 vs. E16 vs. D1 vs. D70	Leg muscle	RNA-seq	Illumina HiSeq 2500	*ALOX5*, *MYH1C*, *FGF8*, *IGF-1*	ECM-receptor, MAPK signaling	[38]
Chicken	Arbor Acres broiler vs. LS chicken (E10, E14, E18, D1, D7, D21, D45)	Pectoralis	RNA-seq	Illumina HiSeq 2500	44-gene network (*RPS29*, *EEF1A2*); *TNFRSF6B* isoforms	Ribosome biogenesis, protein synthesis	[111]
Duck	Pekin (fast-growth) vs. Hanzhong Ma (slow-growth); E17, E21, E27, 6mo.	Pectoralis/leg muscle	RNA-seq	Illumina HiSeq X Ten	*SDC*, *SPP1*, *PAK1*, *MYL9*, *PHGDH*	ECM-receptor interaction, focal adhesion, carbon metabolism	[40]
Chicken	Arbor Acres broiler vs. Zhuanghe Dagu (Local)	Pectoralis & Crureus	RNA-seq	Illumina HiSeq 2000	*EHHADH*, *TECRL*, *GCDH*, *TPI1*; ECM-receptor interaction genes (collagens, laminin, integrin β1)	ECM-receptor interaction, Fatty acid metabolism	[53]

Duck	E17 vs. E21 vs. E27 vs. D180	Pectoralis/leg muscle	RNA-seq	Not specified (Illumina implied)	*CREBL2*, *RHEB*, *GDF6*, *ACTN3*, *RYR3*	Regulation of actin cytoskeleton, oxidative phosphorylation, focal adhesion, ECM-receptor interaction	[39]
Chicken	D28, D84, D112	Leg muscle	RNA-seq	Illumina HiSeq	*FGF16*, *MYH10*, *IGFBP3*, *PHKA1*/*PHKB*/*PHKG1*	Insulin signaling, ECM-receptor interaction, actin cytoskeleton	[37]
Chicken	Gushi (local) vs. Arbor Acres broiler (D42)	Pectoralis	RNA-seq (Ribo-Zero)	Illumina HiSeq 2500	1,649 DEGs (e.g., *LPL*, *FABP5*, *CETP*), 2,540 novel DEGs	Amino acid metabolism, immune pathways	[112]
Turkey	F line (selected) vs. RBC2 (control) at E18, D1, D112	Pectoralis	Microarray	Custom 6K TSKMLO array	↑ *COL6A1*, *β-parvin* (18DEG); Various ECM, apoptosis genes	Calcium signaling, ECM-receptor interaction, tissue development	[113].
Chicken	Broiler vs. Layer (D1, D14, D2, D42, D56)	Pectoralis	Microarray	Affymetrix Chicken Array	*TNNI1*, *MB*, *FHL2*, *CSRP3*, *FABP4*	Muscle development, metabolism, proteolysis	[50]
Goose	E14 vs. E21 vs. E28	Leg muscle	miRNA-seq	Illumina SE50	miR-133a-3p, let-7k-5p, miR-205b	MAPK, TGF-β, Notch signaling	[45]
Chicken	E14 vs. E20 (fast vs. slow growth)	Leg muscle	miRNA-seq	Illumina 2500	gga-miR-146a-3p, gga-miR-2954, *HSPA5*, *PKM2*	Notch, TGF-β, ER stress pathways	[114]
Chicken	Jinghai yellow; fast vs. slow (D300)	Leg muscle	miRNA-seq	Illumina 2500	miR-24-3p, novel_miR_133	BMP signaling, calcium pathway, cell cycle	[115]
Chicken	Oxidative vs. Glycolytic (D140)	Sartorius vs. Pectoralis	RNA-seq	Illumina HiSeq 4000	DElncRNAs (e.g., *XR_003074785.1*); DEcircRNAs (e.g., *novel_circ_004282*)	AMPK, calcium signaling, myoblast differentiation	[116]
Chicken	Hypertrophic (WRR) vs. Lean (XH) broilers (D49)	Pectoralis	RNA-seq	Illumina HiSeq 2000	*lncIRS1*, miR-15a/b/c-5p	IGF1-PI3K/AKT signaling, myogenesis	[15]

Chicken	Oxidative vs. Glycolytic (D140)	Sartorius vs. Pectoralis	RNA-seq + miRNA-seq	Illumina HiSeq 4000/2500	miR-499-5p, *SOX6*, miR-196-5p, *CALM1*	Calcium signaling, PPAR pathway, oxidative phosphorylation	[117]
Duck	E13 vs. E19	Pectoralis	miRNA-seq	Illumina HiSeq 2500	109 DEmiRNAs (e.g., miR-206↑, miR-133a↑, let-7b↑, miR-212-5p↓); 279 novel miRNAs	MAPK signaling, muscle morphogenesis	[43]
Chicken	D42, D98, D154, D210	Pectoralis	RNA-seq + miRNA-seq	Illumina HiSeq 2500	miR-30a-3p, *FOXO3*, *ANKRD1*, miR-148a-3p	FoxO signaling, focal adhesion, ECM-receptor interaction	[118]
Chicken	E11 vs. E16 vs. D1	Leg muscle	miRNA-seq	Illumina HiSeq 2500	miR-222a/*CPEB3*, miR-126-5p/*FGFR3*, miR-133a	TCA cycle, sarcomere organization, MAPK activity	[119]
Chicken	PeM vs. BPR (≤D56)	Pectoralis	miRNA-seq	Illumina TruSeq	miR-206↓, miR-146b-5p↑, let-7b↑	Calcium signaling, NRF2 oxidative response	[120]
Chicken	WRR (fast) vs. XH (slow) (D49)	Pectoralis	miRNA-seq	Solexa	miR-133a/b/c, miR-21, miR-146b-3p	Growth regulation, TGF-β signaling	[121]
Chicken	Broiler vs. Layer (E10)	Pectoralis	miRNA-seq	Solexa	17 DEmiRNAs (e.g., miR-101, miR-15b)	Myogenesis regulation (predicted)	[122]
Chicken	E3, E4, E5	Embryonic somites	miRNA-seq	Solexa	8 novel miRNAs (1 somite-enriched)	N/A	[123]
Pigeon	D3 vs. D14 vs. D25	Pectoralis	RNA-seq	DNBseq	483 DElncRNAs (e.g., *LTCONS_00073284*); 561 DEmRNAs (e.g., *TNNC1*)	Cell cycle, cytoskeleton, ECM-receptor interaction, focal adhesion	[124]
Goose	D3 vs. 3-mon post-hatch	Leg muscle	RNA-seq for lncRNA/mRN, miRNA-seq	Illumina HiSeq 2500/6000	21 DEmiRNAs (e.g., miR-363-3p), 172 DElncRNAs, 1,949 DEmRNAs	MAPK, PPAR, mTOR, Focal adhesion	[125]
Pigeon	D1 vs. D14 vs. D28 vs. 2Years	Pectoralis	RNA-seq	Illumina HiSeq 4000	7,352 DEGs; 4,494 DElncRNAs (e.g., *lncRNA-G12653*)	Cell cycle, muscle development, angiogenesis	[126]

Chicken	E12, E17, D1, D14, D56, D98	Pectoralis	RNA-seq	Illumina HiSeq 4000	2,858 DElncRNAs (e.g. *MSTRG.30304.1*, *MSTRG.31694.1*)	Cell proliferation (Profile 4), Metabolism (Profile 21)	[46]
Chicken	E11, E16, D1	Leg muscle	RNA-seq	Illumina HiSeq 2500	lncRNAs (e.g., *lnc00003323*), *TEAD4*, *DMD*, *FGF13*, *DLK1*	MAPK signaling, embryonic development, cellular proliferation	[127]
Chicken	Gushi vs. Arbor Acres (D42)	Pectoralis	RNA-seq (Ribo-Zero)	Illumina HiSeq 2500	147 DElncRNAs (e.g., *TCONS_00064133*)	MAPK, insulin, calcium signaling pathways	[59]
Chicken	E10, E12, E14, E18	Pectoralis	RNA-seq	Illumina (paired-end 75 nt)	281 intergenic lncRNAs	N/A	[128]
Chicken	Arbor Acres broiler vs. Silky fowl + Insulin (5 IU/kg BW)	Pectoralis	ssRNA-seq	Illumina HiSeq 6000	45 broiler-/29 Silky-specific circRNAs (e.g., *circINSR* downregulated at 15 min)	Insulin signaling, MAPK pathways	[129]
Chicken	Fast vs. slow-growing Bian embryos (E14, E20)	Leg muscle	RNA-seq	Illumina NovaSeq 6000	*novel_circ_0004547*, *novel_circ_0003578*, *novel_circ_0010289*	Wnt signaling, Ubiquitin-mediated proteolysis, Nicotinate metabolism	[5]
Chicken	PEM (fast) vs. SOL (slow) (D49)	Pectoralis	circRNA-seq	Illumina HiSeq 2500	*circPTPN4*, miR-499-3p	AMPK signaling, myogenesis	[130]
Chicken	Fast vs. Slow groups (E14 vs. E20)	Leg muscle	RNA-seq	Illumina NovaSeq 6000	DEcircRNAs (e.g. *novel_circ_0007646*), host genes (*MYH9*, *IGF1R*, *YBX3*)	Adherens junction, myofibril assembly, actin cytoskeleton	[131]
Chicken	Broiler vs. Layer (E10-E19)	Pectoralis	RNA-seq	Illumina (SRA: PRJNA516545)	*circITSN2*, miR-218-5p	Muscle development, ceRNA network	[57]
Chicken	E11 vs. E16 vs. D1	Leg muscle	RNA-seq	Illumina HiSeq 3000	13,377 circRNAs (e.g., *circRBFOX2*, *circSVIL*)	Muscle development, miRNA sponging	[49]
Quail	E9 vs. E14	Leg muscle	Whole transcriptome seq	Illumina HiSeq 4000/Xten	↑*PLIN1*, *SLN*; ↓*MyoD*, *MyoG*	ECM-receptor, PPAR, cell cycle	[132]

Pigeon	E8 vs. E13 vs. D1 vs. D10	Pectoralis	lncRNA-seq + miRNA-seq	Illumina HiSeq 4000/2500	DElncRNAs (e.g. *TCONS_00026594*), miRNAs (e.g. cli-miR-1a-3p), mRNAs (e.g. *FRG1*, *SRC*)	Cell cycle, MAPK signaling, PPAR pathway	[133]

Note: Abbreviations: ACTC1, actin-alpha cardiac muscle-1; ACTN3, actinin alpha 3; ALOX5, arachidonate 5-lipoxygenase; ANKRD1, ankyrin repeat domain-1; APOA1, apolipoprotein AI; CALM1, calmodulin 1; ceRNA, competing endogenous RNA; CETP, cholesteryl ester transfer protein; CLU, clusterin; COL6A1, collagen type-VI-alpha-1-chain; CREBL2, cAMP responsive element binding protein like-2; CSRP3, cysteine and glycine rich protein 3; D, post-hatch day; DEGs, differentially expressed genes; ECM, extracellular matrix; EHHADH, enoyl-CoA hydratase and 3-hydroxyacyl CoA dehydrogenase; EIF4EBP1, eukaryotic translation initiation factor 4E binding protein-1; E, embryonic day; FABP4, fatty acid binding protein 4; FABP5, fatty acid binding protein-5; FDPS, farnesyl diphosphate synthase; FGF16, fibroblast growth factor 16; FGF8, fibroblast growth factor 8; FHL2, four and a half LIM domains-2; FOS, Fos proto-oncogene, AP-1 transcription factor subunit; FOXO3, forkhead box O3; FRG1, FSHD region gene 1 family member B, pseudogene; GDF6, growth differentiation factor-6; IGF-1, insulin like growth factor 1; IGFBP3, insulin like growth factor binding protein 3; MAPK, mitogen-activated protein kinase; MB, myoglobin; MGP, matrix Gla protein; MUSTN1, musculoskeletal, embryonic nuclear protein 1; MyoD, myogenic differentiation 1; MyoG, myogenin; MYH1C, myosin, heavy chain 1C, skeletal muscle; N/A, not available/applicable; NRG1, neuregulin 1; NR1H4, nuclear receptor subfamily 1 group H member 4; PEM/BPR, modern pedigree male broiler vs. Barred Plymouth Rock chicken; PPAR, peroxisome proliferator-activated receptor; PTGS1, prostaglandin-endoperoxide synthase 1; RHEB, Ras homolog enriched in brain; RYR3, ryanodine receptor 3; SLN, sarcolipin; SNRPG, also known as (SNRPGP15) small nuclear ribonucleoprotein polypeptide-G-pseudogene-15; SNRPE, small nuclear ribonucleoprotein polypeptide E; SOX6, SRY-box 6; SRC, SRC proto-oncogene, non-receptor tyrosine kinase; TEAD4, TEA domain transcription factor 4; TGF-β, transforming growth factor beta; TGFB3, transforming growth factor beta 3; TMEM164, transmembrane protein 164; TNNI1, troponin I type 1 (skeletal, slow); ↓, decrease; ↑, increase. Additional abbreviations: Seq. Tech., sequencing technology; Func., functional.

Table S2. Comprehensive Comparison of *In Vivo* RNA Manipulation Techniques in Poultry Muscle Using Viral Vectors.

Poultry Type (age)	Method (dose)	Target RNA	Target Tissue	Duration	Key Findings	Ref.
	**AdV**					
Chicken (D21)	AdV OV ($6×10^6$ μg/mL)	mRNA; *THBS1*	Leg muscles	7D post-injury	Inhibited muscle regeneration, reduced muscle fiber diameter and area, decreased MyHC and Desmin expression	[6]
Chicken (D1/21)	AdV OV ($1.3×10^7$ units/uL)	mRNA; *LDB3*-X, XN1, XN2	GAS/ Tibialis anterior	7-14 D post-injection	*LDB3*-X → ↓atrophy; *LDB3*-XN1/XN1→ ↑atrophy; XN2 → ↑regeneration after $BaCl_2$	[63]
Chicken (D1)	AdV OV ($6×10^6$ PFU)	lncRNA; *lncMGR*	GAS (at multiple points) with BaCl2-induced damage	7D	Promotes muscle fiber hypertrophy in the GAS and soleus and enhances regeneration after $BaCl_2$-induced injury	[67]
Chicken (D21)	AdV OV ($6×10^6$ PFU)	mRNA; *RRM2*	GAS	7D post-$BaCl_2$-induced injury	↓myofiber diameter, ↓muscle regeneration markers (eMyHC, Desmin)	[64]
Chicken (D1)	AdV OV (10^8 titers)	lncRNA; *ZFP36L2-AS*	Bilateral GAS	13D	OV → atrophy; reduced fatty acid β-oxidation & mitochondrial DNA content	[94]
	**LV**					

Chicken	LV OV (1×10^8 IU)	mRNA; *TRA2B*-S/L	GAS	Injected once, analyzed 14D post injection	*TRA2B-S* → ↑ MRFs, muscle fiber size; *TRA2B-L* → opposite outcomes	[69]
Chicken (D1)	LV KD (10^6 titers)	mRNA; *PPARGC1A*	GAS	Injected at 1, 7, 14 D post-hatch; phenotypic assessment at D21	↑glycolysis, ↓oxidative metabolism	[70]
Chicken (D21)	LV OV (1×10^7 IU)	mRNA; *TMEM182*	GAS	9D post-injury	Inhibited regeneration (↑ necrosis, ↓ regeneration markers), induces atrophy (↓ fiber diameter)	[92]
Chicken (D1)	LV KD/OV (N/A)	mRNA; *Fmod*	Leg muscle	9D	KD: ↓ fiber area/diameter, ↑ atrophy genes; OV: ↑ hypertrophy	[73]
Japanese Quail (D42, D700)	LV OV (10uL, 2.1x10^7 TU/mL)	mRNA; eGFP (reporter)	Patagialis muscle	Expression analyzed 14-21 D post-injection	Effective gene delivery in aged, hypertrophying muscle; polybrene increases expression by ~41%	[68]
Chicken (D12)	LV KD/OV (10^{-9} TU/mL)	miRNA; miR-22-3p	Wing vein	7D	Modulates KLF3/6/12 in liver but not pectoral muscle	[88]
Chicken (D1)	LV OV (10^6 TU)	lncRNA; *MYH1G-AS*	GAS	3 doses at D1, 7, 14; analyzed at 21D	↑ fast-twitch fibers, muscle atrophy via FGF18	[134]
Chicken (D1)	LV KD (10^6 titers)	lncRNA; *ZFP36L2-AS*	Bilateral GAS	13D	↑muscle mass, ↑slow-twitch fibers	[94]

Chicken (D7)	LV OV (3x10^6 TU)	lncRNA; *SMARCD3-OT1*	Left GAS (Treated) vs. right GAS (Control)	2 doses at D 7 and 14; analyzed 14D after the initial injection	↑ muscle mass, hypertrophy, fast-twitch fiber genes (*MYH1*↑, *MYH7*↓); ↑ LDH activity	[95]
Chicken (D1)	LV KD/OV (1x10^6 TU/mL)	lncRNA; *LncEDCH1*	Left GAS (Treated) vs. right GAS (Control)	3 doses at D1, 4 and 8; analyzed 14D after the initial injection	↓ *lncEDCH1* → ↑ atrophy, ↓ FAO; OV → ↑ slow-twitch fibers	[17]
Chicken (D1)	LV OV/KD (1x10^6 TU/mL)	lncRNA; *FKBP1C*	GAS	3 doses at D 1, 5 and 9; analyzed on D14 after the initial injection	↑ slow-twitch fibers, ↓ glycogen; Altered LDH/SDH activity; Fiber hypertrophy	[89]
Chicken (D1)	LV OV/KD (10^6 TU)	lncRNA; *SMUL*	GAS	2 doses at D1 and 7; analyzed after 13D	*SMUL*↑ → atrophy, fast-twitch fibers; *SMUL*↓ → hypertrophy, slow-twitch fibers	[93]
Chicken (D1)	LV OV/KD (1 × 10^8 IU/mL)	lncRNA; *lncIRS1*	Breast muscle	3 doses at D1, 3, and 5; analyzed on D9 after the initial injection	↑ muscle mass/fiber CSA; ↓ atrophy genes (*Atrogin-1*)	[15]
Chicken (D1)	LV OV (1×10^7 TU)	circRNA; *circAGO3*	Breast muscle	2 doses at D1 and D7; analyzed on D14 after the initial injection	↑ muscle atrophy, ↓ myofiber area, ↑ inflammation (*TNF-α*, *IL-1β*)	[72]

Chicken (D1)	LV KD (1 × 10^7 TU per injection)	circRNA; *circMEF2A1/2*	Breast muscle	2 doses at D1 and D8; tissue harvest (14D post-first injection)	↓ breast muscle mass, ↓ muscle/body weight ratio, ↓ myofiber CSA	[80]
Chicken (D1)	LV KD (1 × 10^8 TU)	circRNA; *circGPD2*	Breast muscle (injected at both sides with 5 injection sites)	14D	↓ breast muscle mass, ↓ myofiber size, ↓ MyoG/MyoD	[71]
	**RCAS**					
Chick embryo (E2-E9)	*In ovo* electroporation; RCAS-R	mRNA; *MSTN, FST, SMAD7*	Somite / dermomyotome (trunk & limb muscle progenitors)	Short-term (3.5D) & long-term (7.5D) analysis	*MSTN* OE → ↑ differentiation ($MyHC^+$), ↓ progenitor pool ($Pax7^+$). *MSTN* inhibition → ↑ progenitor pool, ↓ differentiation	[30]
Chick embryo (≤E2.3)	RCAS-R OV ($2×10^8$ CFU/mL)	mRNA; dn*FGFR1*	Somites 26–31 and hind limb infection	E8,12,19 (up to 14D post-infection)	~30% ↓ muscle mass, ~50% ↓ myofiber density, loss of myoblasts/fibroblasts, disrupted fiber organization.	[76]
Chick embryo (E4)	RCAS-R OV ($2×10^4$ CFU)	mRNA; *FGF5*	Hind limb bud	7D post-infection	Smaller muscle masses, reduced MyoD/MyHC expression	[77]
Chick embryos (stage 10-11/ 12–14)	RCAS-R cell injection	mRNA; *BMP-4*/*Wnt-1*	Paraxial mesoderm/neural tube	1–2 D	BMP-4 → ↑Wnt-11; Wnt-1 → ectopic myotome patterning	[74]

Note: Abbreviations: AdV, adenovirus; BMP-4, bone morphogenetic protein 4; CFU, colony forming units; circRNA, circular RNA; CSA, cross-sectional area; D, post-hatch

day; E, embryonic day; FGF5, fibroblast growth factor 5; Fmod, fibromodulin; GAS, gastrocnemius muscle; IU, infectious units; KD, knockdown; lncRNA, long non-coding RNA; LV, lentivirus; miRNA, microRNA; mL, milliliter; N/A, not available/applicable; OE, overexpression; OV, overexpression vector; PFU, plaque-forming units; PPARGC1A, PPARG coactivator-1-alpha; RCAS-R, RCAS retrovirus; RRM2, ribonucleotide reductase regulatory subunit-M2; SMAD7, SMAD family member 7; THBS1, thrombospondin 1; TMEM182, transmembrane protein 182; TU, transduction unit; Wnt-1, Wnt family member 1; →, leads to; ↓, decrease; ↑, increase.

Table S3. Transcriptomic profiles and targeted studies of poultry myopathies.

Poultry Type	Myopathy	Comparison /Age (D)	RNA Type	Main Analysis Method	Key Dysregulated RNAs	Implicated Pathways/Functions	*In Vivo* Validation?	Ref.
Broiler chicken	WS	Mild WS vs. Normal at D35	mRNA	RNA-seq (Illumina NextSeq 2000)	↑*PDK4*, *CEBPD*, *COL4A5*; ↓*METTL21EP*, *DUSP8*	Muscle development, lipid metabolism, collagen	None	[135]
Broiler chicken	WB	WB vs. Normal at D23	mRNA	Spatial Transcriptomics (Visium/Illumina NextSeq 2000)	↑ *FABP4*, *LPL*, *PLIN1*, *CTSB*, *COL12A1*	Lipid metabolism, lysosomal activity, ECM remodeling	None	[98]
Broiler chicken	WB	WB vs. Normal at D47	mRNA	RNA-seq (GRCg7b)	↑*WFIKKN1*, *GFPT*1/2, *UGDH*; ↓*PPARGC1A*/*B*, *FOXO1*, *NRF1*	Mitochondrial dysfunction, HBP shift, hypertrophy	None	[103]
Broiler chicken	WB	WB vs. Normal at D45	mRNA	RNA-seq + WGCNA	↑*TGFB*1/2/3, *MYD88*, *FOS*; ↓*GAS6*, *GLRX*	TGF-β, TLR signaling, autophagy	None	[104]
Broiler chicken	WB	Severe vs. Mild WB at D36	mRNA	RNA-seq (NovoSeq 6000)	↑*SDC4*, *MMP2/9*, *COL1A1*, *LOX*, *LUM*; ↓*SDC2*	ECM remodeling, fibrosis, inflammation	None	[97]
Broiler chicken	WB	WB vs. Normal (D21/42/56)	mRNA	RNA-seq (single-end 75nt)	↑ *OTUD1*, *SACS*, *CXCR4*, *HMOX1*, *ALDOB*, *GPD1L2*; ↓ *PYGB*, *MGAM*	Hypoxia response, ubiquitin proteolysis, glycolysis, ECM remodeling	Yes (Triglyceride kinetics)	[102]
Broiler chicken	WB	U, P, A severity classes at D47/48	mRNA	nCounter MAX	↑*ESM1*, *TLR2-2*, *TLR4*; ↑↓*LPL* (U vs. P/A); ↓*DGAT2*	Vascular dysfunction, lipid metabolism	None	[10]
Broiler chicken	WB	WB vs. Normal at D42	mRNA	Microarray + ddPCR	↑*AKR1D1*, *COL12A1*; ↓*GFRA4*	Glucose/lipid metabolism, apoptosis	None	[99]

Broiler chicken	WB	WB vs. Normal at D49	mRNA	Microarray + ddPCR	↑*THBS2*, *ACTN1*, *ILK*; ↓*MYLK4*, *ITGB5*	Focal adhesion, actin cytoskeleton, ECM	None	[99]
Broiler chicken	WB	WB vs. Normal at D42	mRNA	qPCR/Western Blot	↑*IL-1β*, *TNF-α*, *IL-6*, *TLR2/4/5*, iNOS, *COX-2*, PTGEs ↓*IL-4*, *IL-10*	TLR/NF-κB signaling, inflammation, fibrosis	Tissue assays	[11]
Broiler chicken	WS	Moderate WS vs. Normal at D42	mRNA	qPCR	↑ *CA2*, *CSRP3*, *PLIN1;* ↓ *CALM2*, *DNASE1L3*, *MYLK2*	Calcium signaling disruption, myogenic regulation, lipid metabolism, DNA fragmentation/apoptosis	None	[136]
Broiler chicken	WB	WB vs. Normal at D45	mRNA	RNA-seq (Illumina HiSeq 2500) + Machine Learning	↑*NUP43*; ↓*KPNA7*, *RPL19*	TGF-β signaling, ribosome biogenesis	None	[21]
Broiler chicken	WS	Severe vs. Mild WS at D49	mRNA	Microarray (8×60K Agilent chip)	↑ *NOS3*, *PLCB*, *CAMK2D*; ↓ *CALM*, *PPP2R2B*	Calcium signaling, oxidative stress, apoptosis	None	[137]
Broiler chicken	WS/WB	WS and WB vs. Normal at D42	mRNA	qPCR	↑ *FN1*, *NCAM*, *MYH15*, *TGFB1*, *CTGF*; ↓ *MYH1E*, *MYH13*; *PPARG* (↑ WB & WS severity-dependent)	Fibrosis (TGF-β), adipogenesis, muscle regeneration, mitochondrial dysfunction	None	[100]
Broiler chicken	WS/WB	WS/WB vs. Normal at D180	mRNA	ddPCR	↑*HIF1A*, *GSTM2*; ↓*LDHA*, *PFKFB4* (in WB)	Hypoxia response, glycolysis, oxidative stress	None	[101]
Broiler chicken	WS/WB	FG-WSWB vs. FG-C at D42	mRNA	Microarray (8×60K Agilent chip)	↑ *FN1*, *COL6A3*, *MYH15*; ↓ *MYH1E*	Fibrosis, hypoxia response, regeneration	None	[96]
Broiler chicken	WS	Severe WS vs. Normal at D42	mRNA	RNA-seq (Illumina HiSeq2500)	↑ *HIF1A*, *EDNRA*, *CAMK2A*, *CRH* ↓ *FGF1*, *ROCK2*, *RYR1*	Hypoxia response, angiogenesis failure, calcium signaling, apoptosis	None	[20]
Broiler chicken	WB	Male vs. Female	mRNA	RNA-seq (Illumina	↑ *FABP4*, *PLIN1*, *LPL*, *CHAC1*; ↓ *CD24*, *TNNT1*	Lipid metabolism, oxidative stress, antiangiogenesis	None	[105]

		(unaffected muscle), D21		HiSeq 2500)				
Broiler chicken	WB	WB vs. Normal at D14	mRNA	RNA-seq (Illumina HiSeq 2500)	↑*PPARG*, *FABP4*, *LPL*, *CIDEA*; ↓*BRSK2*	Lipid metabolism, PPARγ signaling, ER stress	None	[106]
Broiler chicken	WB	Early WB (D14–28) vs. Normal	mRNA	RNA-seq (Illumina HiSeq 2500)	↑ *Fmod*, *COL12A1*, *C3*, *SPP1* ↓ *MTMR14*, *SRL*, *SYPL2*	ECM remodeling, complement activation, calcium signaling, lipid metabolism	None	[18]
Broiler chicken	WB/WS	Affected vs. Normal at *D52	mRNA, miRNA, snoRNA	Microarray (Affymetrix GeneChip)	↑ *MB*, *CRYAB*, *ATP2A2*, miR-205a; ↓ *PGAM*, *GPI*, miR-196b	Calcium signaling, oxidative stress, ECM remodeling	None	[14]
Broiler chicken	WB	WB vs. Normal at D47	mRNA	RNA-seq (Illumina HiSeq)	↑*COL1A1*, *CA3*, *PLOD2*; ↓*MYH1E*, *FGB*	ECM remodeling, hypoxia, calcium homeostasis	None	[13]
Broiler chicken	WB	WB vs. Normal at D42	miRNA	RNA-seq + miRNA-seq (Illumina NovaSeq/ HiSeq)	↑*ITGAV*, *TGFB1*, *MMP9*, miR-21-3p ↓*GPI*, *LDHA*, miR-183, miR-96-5p	ECM-integrin interaction, TGF-β signaling, inflammation, suppressed glycolysis/amino acid metabolism, miRNA-mRNA networks	None	[19]
Broiler chicken	WB & WS	WB/WS vs. Normal at D28	miRNA	miRNA-seq (Illumina NextSeq 2000)	↑miR-155, miR-29; ↓miR-122, miR-200b-3p	Autophagy, insulin signaling, ER protein processing	None	[138]
Broiler chicken	WB	WB vs. Normal at D42	miRNA	miR-NA-seq (Illumina HiSeq 2500)	↑miR-155, miR-29c; ↓miR-133a	Energy metabolism, fibrosis, vascular development	None	[139]
Broiler chicken	WB	WB vs. Normal at D47	mRNA	RNA-seq + WGCNA	↑*ACTN1*, *ZYX*; ↓*CARNS1*, *GOT2*	Focal adhesion, antioxidant depletion, energy metabolism	None	[140]

Note: Abbreviations: A, markedly affected; ACTN1, actinin, alpha 1; AKR1D1, aldo-keto reductase family 1 member D1; ALDOB, aldolase, fructose-bisphosphate-B; CA2, carbonic anhydrase-2; CA3, carbonic anhydrase 3B; CALM2, calmodulin 2; CAMK2A, calcium/calmodulin dependent protein kinase II alpha; CAMK2D,

calcium/calmodulin dependent protein kinase-II-delta; CARNS1, carnosine synthase 1; CD24, CD24 molecule; CEBPD, CCAAT/enhancer binding protein delta; CHAC1, ChaC glutathione-specific gamma-glutamylcyclotransferase-1; COL1A1, collagen type-I-alpha-1-chain; COL12A1, collagen type-XII-alpha-1-chain; CSRP3, cysteine and glycine rich protein 3; CTGF, also known as CCN2 (cellular communication network factor 2); OTUD1, OTU deubiquitinase 1; CTSB, cathepsin-B; CXCR4, C-X-C motif chemokine receptor-4; D, post-hatch day; ddPCR, droplet digital PCR; DNASE1L3, deoxyribonuclease-1-like 3; ECM, extracellular matrix; ESM1, endothelial cell specific molecule 1; FABP4, fatty acid binding protein-4; FG-C, normal; FG-WSWB, severely affected by both white striping and wooden breast; FGB, fibrinogen beta chain; FN1, fibronectin 1; Fmod, fibromodulin; GLRX, glutaredoxin; GPI, glucose-6-phosphate isomerase; GPD1L2, glycerol-3-phosphate dehydrogenase-1; HIF1A, hypoxia inducible factor 1 alpha subunit; HMOX1, heme oxygenase-1; IL-4, interleukin 4; IL-10, interleukin 10; ITGAV, integrin subunit alpha V; KPNA7, karyopherin subunit alpha 7; LDHA, lactate dehydrogenase A; LPL, lipoprotein lipase; MGAM, maltase-glucoamylase (alpha-glucosidase); MMP9, matrix metallopeptidase 9; MTMR14, myotubularin related protein 14; MYD88, MYD88 innate immune signal transduction adaptor; MYH1E, myosin, heavy chain 1E, skeletal muscle; MYLK2, myosin light chain kinase-2; NOS3, nitric oxide synthase-3; NRF1, nuclear respiratory factor-1; NUP43, nucleoporin 43; P, partially affected; PDK4, pyruvate dehydrogenase kinase 4; PFKFB4, 6-phosphofructo-2-kinase/fructose-2,6-biphosphatase 4; PLCB, phospholipase C-beta-1; PLOD2, procollagen-lysine,2-oxoglutarate 5-dioxygenase 2; PPARG, peroxisome proliferator-activated receptor gamma; PPP2R2B, protein phosphatase-2-regulatory-subunit-Bbeta; PYGB, phosphorylase, glycogen-brain; RPL19, ribosomal protein-L19; SACS, SACS sacsin molecular chaperone; SDC2, syndecan-2; SPP1, secreted phosphoprotein 1; SRL, sarcalumenin; SYPL2, synaptophysin like 2; TGFB1, transforming growth factor beta 1; THBS2, thrombospondin 2; TNNT1, troponin T1, slow skeletal type; U, unaffected pectoralis; UGDH, UDP-glucose 6-dehydrogenase; WB, wooden breast; WFIKKN1, WAP, follistatin/kazal, immunoglobulin, kunitz and netrin domain containing-1; WGCNA, weighted gene co-expression network analysis; WS, white striping; ZYX, zyxin; ↓, decrease; ↑, increase. Method clarification: nCounter MAX (NanoString) is a targeted digital counting method for transcript quantification. *The age (D52) for reference [14] is as reported in reference [12].
